# Machine learning to optimize the diagnostic performance of natriuretic peptides for acute heart failure across age groups

**DOI:** 10.1093/eschf/xvaf006

**Published:** 2026-01-08

**Authors:** Daniel Perez Vicencio, Dimitrios Doudesis, Alexander J F Thurston, Camille Chenevier-Gobeaux, Yann-Erick Claessens, Pedro Lopez Ayala, Maria Belkin, Desiree Wussler, Christopher deFilippi, Stephen Seliger, Gordon Moe, Carlos Fernando, Antoni Bayes-Genis, Yigal Pinto, Hanna K Gaggin, Jan C Wiemer, Martin Möckel, Joost H W Rutten, Luna Gargani, Nicola R Pugliese, Christopher Pemberton, Irwani Ibrahim, Alfons Gegenhuber, Thomas Mueller, Michael Neumaier, Michael Behnes, Ibrahim Akin, Michele Bombelli, Guido Grassi, Peiman Nazerian, Giovanni Albano, Philipp Bahrmann, A Mark Richards, John J V McMurray, Christian Mueller, James L Januzzi, Nicholas L Mills, Kuan Ken Lee

**Affiliations:** British Heart Foundation (BHF) Centre for Cardiovascular Science, University of Edinburgh, Edinburgh EH16 4SA, UK; Usher Institute, University of Edinburgh, Edinburgh, UK; British Heart Foundation (BHF) Centre for Cardiovascular Science, University of Edinburgh, Edinburgh EH16 4SA, UK; Usher Institute, University of Edinburgh, Edinburgh, UK; British Heart Foundation (BHF) Centre for Cardiovascular Science, University of Edinburgh, Edinburgh EH16 4SA, UK; Department of Biochemistry, Cochin Hospital, Assistance Publique-Hopitaux de Paris, Paris, France; Department of Emergency Medicine, Princess Grace Hospital Center, Monaco, Principalty of Monaco; Cardiovascular Research Institute of Basel, Department of Cardiology, University Hospital Basel, Basel, Switzerland; Cardiovascular Research Institute of Basel, Department of Cardiology, University Hospital Basel, Basel, Switzerland; Cardiovascular Research Institute of Basel, Department of Cardiology, University Hospital Basel, Basel, Switzerland; Department of Cardiology, Vancouver General Hospital, University of British Columbia, Vancouver, BC, Canada; Division of Cardiology, University of Maryland School of Medicine, Baltimore, MD, USA; Division of Nephrology, University of Maryland School of Medicine, Baltimore, MD, USA; Department of Medicine, University of Toronto, St Michael’s Hospital, Toronto, ON, Canada; Department of Medicine, University of Toronto, St Michael’s Hospital, Toronto, ON, Canada; Cardiology Department, Heart Institute, Hospital Universitari Germans Trias I Pujol, CIBERCV, Badalona, Spain; Department of Experimental Cardiology, Amsterdam Cardiovascular Sciences, University of Amsterdam, Amsterdam, The Netherlands; Department of Medicine, Harvard Medical School, Boston, MA, USA; Division of Cardiology, Massachusetts General Hospital, Boston, MA, USA; BRAHMS, Thermo Fisher Scientific, Hennigsdorf, Germany; Department of Emergency and Acute Medicine with Chest Pain Units, Charité—Universitätsmedizin Berlin, Campus Mitte and Virchow, Berlin, Germany; Department of Internal Medicine, Radboud University Medical Center, Nijmegen, The Netherlands; Department of Surgical, Medical and Molecular Pathology and Critical Care Medicine, University of Pisa, Pisa, Italy; Department of Clinical and Experimental Medicine, University of Pisa, Pisa, Italy; Christchurch Heart Institute, University of Otago, Christchurch, New Zealand; Emergency Medicine Department, National University Hospital, Singapore; Department of Internal Medicine, Krankenhaus Bad Ischl, Bad Ischl, Austria; Department of Laboratory Medicine, Hospital Voecklabruck, Voecklabruck, Austria; Institute for Clinical Chemistry, Faculty of Medicine Mannheim, University Medical Centre Mannheim, University of Heidelberg, Mannheim, Germany; First Department of Medicine, Faculty of Medicine Mannheim, University Medical Centre Mannheim, University of Heidelberg, Mannheim, Germany; First Department of Medicine, Faculty of Medicine Mannheim, University Medical Centre Mannheim, University of Heidelberg, Mannheim, Germany; Clinica Medica, University of Milano—Bicocca, Monza, Italy; Clinica Medica, University of Milano—Bicocca, Monza, Italy; Department of Emergency Medicine, Azienda Ospedaliero-Universitaria Careggi, Florence, Italy; Department of Emergency Medicine, Azienda Ospedaliero-Universitaria Careggi, Florence, Italy; Institute for Biomedicine of Aging, Friedrich-Alexander-University, Nuremberg, Germany; Christchurch Heart Institute, University of Otago, Christchurch, New Zealand; Cardiovascular Research Institute, National University Heart Centre Singapore, Singapore; BHF Cardiovascular Research Centre, University of Glasgow, Glasgow, UK; Cardiovascular Research Institute of Basel, Department of Cardiology, University Hospital Basel, Basel, Switzerland; Department of Medicine, Harvard Medical School, Boston, MA, USA; Division of Cardiology, Massachusetts General Hospital, Boston, MA, USA; British Heart Foundation (BHF) Centre for Cardiovascular Science, University of Edinburgh, Edinburgh EH16 4SA, UK; Usher Institute, University of Edinburgh, Edinburgh, UK; British Heart Foundation (BHF) Centre for Cardiovascular Science, University of Edinburgh, Edinburgh EH16 4SA, UK

**Keywords:** Acute heart failure, Natriuretic peptides, NT-proBNP, Machine learning

## Abstract

**Background and Aims:**

N-terminal pro-B-type natriuretic peptide (NT-proBNP) concentrations are influenced by age, which may influence the diagnostic performance of this peptide. Machine learning approaches incorporating NT-proBNP and age as continuous measures may have improved diagnostic performance.

**Methods:**

We pooled individual patient-level data for 10 369 patients [median age 73 years (25th–75th percentile: 59–82)] with suspected acute heart failure across fourteen studies. The diagnostic performance of guideline-recommended NT-proBNP thresholds (uniform rule-out threshold of 300 pg/mL and age-stratified rule-in thresholds of 450, 900, and 1800 pg/mL for patients <50, 50–75, and >75 years, respectively) and the Collaboration for the Diagnosis and Evaluation of Heart Failure (CoDE-HF) machine learning model were evaluated using random effects meta-analysis across age groups.

**Results:**

Overall, 43.9% (4549/10 369) of patients had an adjudicated diagnosis of acute heart failure. The negative predictive value (NPV) of the rule-out threshold of 300 pg/mL was lower in older patients [NPV 88.7% (confidence interval (CI) 84.2–92.1%) in patients ≥80 years vs 98.9% (97.6–99.5%) <50 years]. Conversely, the positive predictive value (PPV) of age-stratified rule-in thresholds was lower in younger patients [PPV 62.0% (56.2–67.5%) in those <50 years vs 79.6% (70.7–86.3%) ≥80 years]. CoDE-HF was more accurate than guideline-recommended thresholds across all age groups, with NPV and PPV ranging from 96.4% to 99.5% (93.8–99.8% CIs) and 81.1% to 84.2% (74.7–90.4% CIs), respectively.

**Conclusion:**

The diagnostic performance of guideline-recommended thresholds of NT-proBNP varies significantly with age. A decision-support tool incorporating NT-proBNP with age as a continuous variable provides a more consistent and accurate approach.

## Graphical Abstract

**Graphical Abstract:**
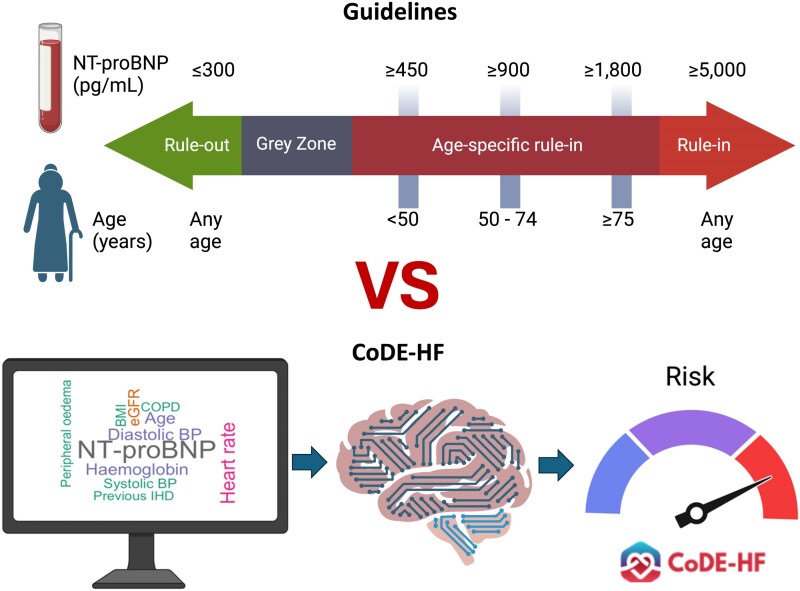

## Introduction

Acute heart failure is a leading cause of hospitalization in elderly patients, and the incidence is expected to rise substantially due to ageing populations.^1,2^ However, diagnosing acute heart failure in older patients is more challenging because they often present with more comorbidities which can complicate the clinical presentation.^3^ Many of the signs and symptoms of acute heart failure can be caused by other life-threatening conditions that are more prevalent in older than younger patients. Nevertheless, an accurate and timely diagnosis is crucial because delays in diagnosis and provision of evidence-based therapies are associated with increased length of hospital stay and mortality.^4,5^

Current guidelines recommend N-terminal pro-B-type natriuretic peptide (NT-proBNP) testing to aid in diagnosing acute heart failure.^6,7^ However, NT-proBNP concentrations increase with advancing age, presumably due to accumulating comorbidities and structural heart disease; this may impact the diagnostic performance of NT-proBNP for acute heart failure in older individuals.^8^ Guidelines now recommend age-stratified NT-proBNP rule-in thresholds and a uniform threshold of 300 pg/mL to exclude acute heart failure.^9–14^ Given the challenges posed by using dichotomous thresholds for diagnostic biomarkers, we recently developed and validated a decision-support tool for acute heart failure called CoDE-HF (Collaboration for the Diagnosis and Evaluation of Heart Failure) that uses a machine learning algorithm to combine NT-proBNP concentrations and age as continuous variables.^15^

In this study, we aimed to evaluate the diagnostic performance of guideline-recommended NT-proBNP thresholds and the CoDE-HF decision-support tool for acute heart failure across age groups.

## Methods

### Study population

We performed a systematic review to identify all studies evaluating the diagnostic performance of NT-proBNP in patients with suspected acute heart failure, following a pre-specified protocol registered in PROSPERO (International Prospective Register of Systematic Reviews; CRD42019159407). A detailed description of our systematic review methodology has been published previously.^15^ We identified studies that enrolled patients aged ≥18 years evaluated for acute medical presentations with suspected acute heart failure, measured NT-proBNP concentrations, and adjudicated the diagnosis of acute heart failure using an acceptable reference standard (Supplementary Table S1). In most cohorts, adjudicators were blinded to NT-proBNP concentrations to minimize incorporation bias. Authors of all eligible studies were contacted for individual patient-level data. A total of 14 studies provided individual patient-level data in 10 369 patients for our analysis (Supplementary Tables S2 and S3).

### CoDE-HF score

A detailed description of the development and validation of CoDE-HF (https://decision-support.shinyapps.io/code-hf/) has been published previously.^15^ CoDE-HF uses an extreme gradient boosting (XGBoost) algorithm to compute a value (0–100) that corresponds to an individual patient’s probability of acute heart failure.^16,17^ The variables included in CoDE-HF are NT-proBNP, age, estimated glomerular filtration rate, haemoglobin, body mass index, heart rate, blood pressure, peripheral oedema, chronic obstructive pulmonary disease, and ischaemic heart disease.

### Statistical analyses

We calculated meta-estimates with 95% confidence intervals of the sensitivity, specificity, negative predictive value (NPV), and positive predictive value (PPV) of the guideline-recommended NT-proBNP rule-out threshold of 300 pg/mL and age-specific rule-in thresholds (450, 900 and 1800 pg/mL for patients aged <50, 50–75, and >75 years, respectively) across 10-year age groups (<50, 50–59, 60–69, 70–79, ≥80 years) and subsequently across age as continuous variable. The diagnostic performance of the CoDE-HF rule-in and rule-out thresholds were also calculated across the same age groups. Estimates were calculated within each study and then pooled across studies in a binomial-normal random effects model using the DerSimonian and Laird method.^18^ Overall discrimination was evaluated using receiver operating curve analyses. We subsequently performed a sensitivity analysis restricted to patients who were not previously known to have heart failure (Supplementary Table S4). All data analysis was conducted in R (version 4.3.0, R Foundation for Statistical Computing).

## Results

### Study population

Overall, individual patient-level data on 10 369 patients with suspected acute heart failure [median age 73 years (p25–p75 59–82); 53.3% male] were harmonized from 14 cohorts across 13 countries.^19–32^ The median NT-proBNP concentration was 1182 pg/mL (191–4737 pg/mL) (*Table 1*). All studies were conducted in the Emergency Department, except for one that included patients hospitalized in the cardiology and pulmonology departments. In total, 4549 (44%) patients had an adjudicated diagnosis of acute heart failure with a median prevalence of 46% (31%–54%) across studies.

**Table 1:** Baseline characteristics of patients stratified by age groups

	All participants (*n* = 10 369)	<50 (*n* = 1360)	50–59 (*n* = 1300)	60–69 (*n* = 1787)	70–79 (*n* = 2457)	≥80 (*n* = 3465)
**Male sex**	5531 (53)	692 (51)	766 (59)	1127 (63)	1374 (56)	1572 (45)
**Ethnicity**						
**Black**	845 (15)	284 (30)	211 (23)	156 (14)	115 (8.0)	79 (6.2)
**White**	4112 (72)	423 (45)	489 (54)	833 (73)	1208 (84)	1159 (91)
**Other**	743 (13)	227 (24)	208 (23)	152 (13)	116 (8.1)	40 (3.1)
**Past medical history**						
**Previous heart failure**	3119 (33)	166 (12)	279 (22)	552 (31)	921 (38)	1201 (48)
**Ischaemic heart disease**	2953 (32)	96 (7.2)	264 (21)	569 (33)	974 (41)	1050 (42)
**Diabetes**	2398 (27)	183 (14)	352 (28)	560 (33)	758 (32)	545 (23)
**Hypertension**	5071 (59)	305 (25)	606 (53)	941 (60)	1523 (68)	1696 (72)
**Hyperlipidaemia**	2269 (41)	130 (16)	295 (38)	501 (48)	755 (54)	588 (41)
**Current smoker or ex-smoker**	2458 (41)	470 (47)	444 (50)	523 (47)	615 (40)	406 (29)
**Asthma**	770 (19)	275 (35)	165 (23)	137 (16)	117 (12)	76 (9.4)
**Chronic obstructive pulmonary disease**	2117 (29)	107 (10)	258 (26)	468 (35)	676 (36)	608 (30)
**Atrial fibrillation**	1701 (21)	53 (4.9)	118 (10)	260 (17)	548 (25)	722 (33)
**Chronic kidney disease**	1215 (19)	32 (3.2)	75 (8.3)	192 (16)	343 (21)	573 (34)
**Body mass index, kg/m^2^**	28 (7)	29 (9)	30 (8)	29 (7)	28 (6)	25 (5)
**<25**	3062 (39)	475 (41)	327 (31)	503 (33)	718 (34)	1039 (51)
**25–29**	2473 (31)	279 (24)	315 (30)	459 (31)	742 (36)	678 (33)
**≥30**	2317 (30)	415 (36)	412 (39)	540 (36)	629 (30)	321 (16)
**Physiological parameters**						
**Heart rate, beats per minute**	92 (24)	95 (21)	93 (23)	93 (24)	90 (25)	90 (24)
**Systolic blood pressure, mmHg**	140 (28)	134 (25)	140 (28)	141 (29)	141 (28)	141 (29)
**Diastolic blood pressure, mmHg**	80 (17)	82 (17)	84 (17)	81 (17)	78 (17)	77 (17)
**Clinical haematology and biochemistry**						
**Haemoglobin, g/dL**	13.11 (2.11)	13.76 (2.10)	13.57 (2.08)	13.37 (2.05)	12.96 (2.06)	12.50 (2.02)
**eGFR, mL/min/1.73 m^2^**	68 (31)	100 (32)	83 (29)	73 (30)	62 (26)	53 (24)
**NT proBNP, pg/mL**	1182 [191–4737]	64 [20–376]	256 [60–1640]	776 [149–3637]	1435 [370–4850]	3136 [1020–8463]
**Adjudicated diagnosis of heart failure**	4549 (44)	218 (16)	366 (28)	703 (39)	1235 (50)	2027 (58)

Values are median [interquartile range], *n* (%) or mean ± SD. eGFR, estimated glomerular filtration rate; NT-proBNP, N-terminal pro-B-type natriuretic peptide.

The prevalence of comorbidities increased considerably with age (*Table 1*). Patients ≥80 years had a four times higher prevalence of prior heart failure compared with those <50 years (48% vs 12%). Older patients also had a higher prevalence of ischaemic heart disease, hypertension, atrial fibrillation and chronic kidney disease. NT-proBNP concentrations were higher in older patients [median of 64 (20–376) pg/mL in patients <50 vs 3136 (1020–8463) in those ≥80 years old]. Older patients were also more likely to have an adjudicated diagnosis of acute heart failure (16% in patients <50 vs 58% in those ≥80 years). Similar findings were observed in a sensitivity analysis restricted to patients without a prior heart failure diagnosis (Supplementary Table S4).

### Diagnostic performance of guideline-recommended thresholds

**Figure 1:**
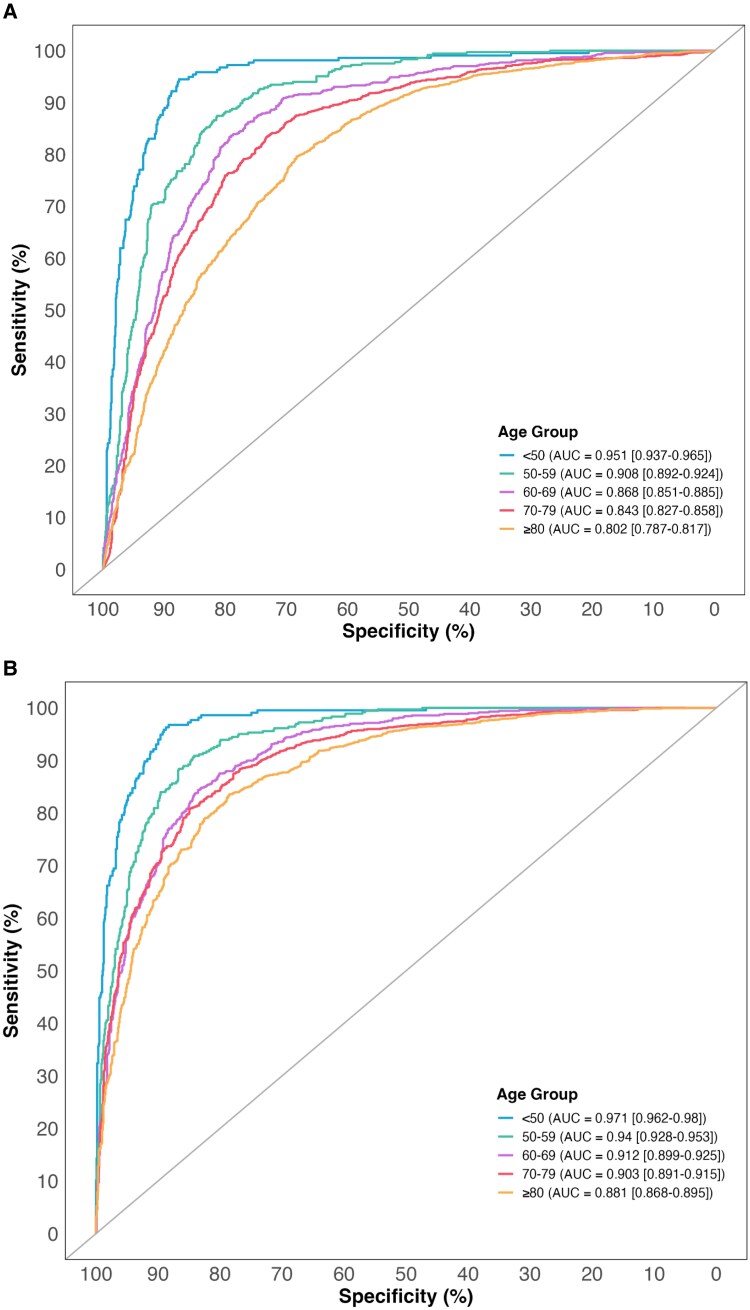
Discrimination of N-terminal-pro B-type natriuretic peptide and CoDE-HF for the diagnosis of acute heart failure. (A) Receiver operator curve of N-terminal-pro B-type natriuretic peptide stratified by age groups. (B) Receiver operator curve of CoDE-HF stratified by age groups

**Figure 2:**
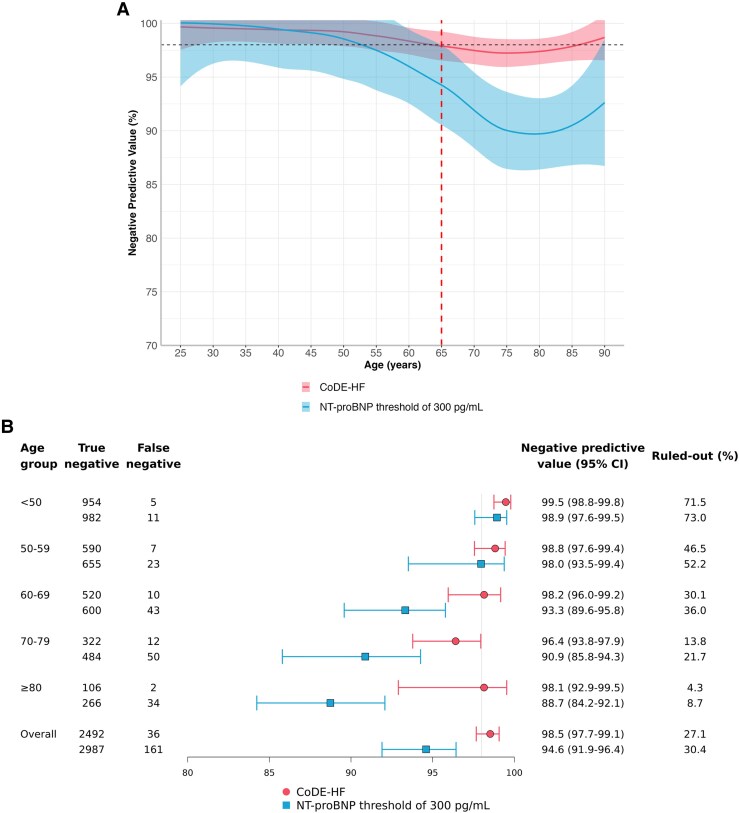
Negative predictive value of N-terminal-pro B-type natriuretic peptide rule-out threshold of 300 pg/mL and CoDE-HF low-probability score. (A) Negative predictive value across age as a continuous variable. (B) Forest plot of negative predictive value across patient age groups

Overall, the discrimination by NT-proBNP alone for the diagnosis of acute heart failure declined with age from an area under receiver operator curve (AUC) of 0.951 (0.937–0.965) in patients <50 to an AUC of 0.802 (0.787–0.817) in those ≥80 years (*Figure 1A*). The guideline-recommended rule-out threshold of 300 pg/mL had a sensitivity of 96.8% (94.6%–98.1%) and an NPV of 94.6% (91.9%–96.4%) in all patients (Supplementary Table S5). Across the age continuum, the NPV of an NT-proBNP threshold of 300 pg/mL decreased below 98% in patients older than 65 years (*Figure 2A*). NPV was 98.9% (97.6%–99.5%) in those <50 years but decreased to 88.7% (84.2%–92.1%) in those ≥80 years (*Figure 2B*). Sensitivity was lower in younger patients [95% (91.1%–97.2%) <50 years] and improved with age [98.4% (97.2%–99%) ≥80 years] (Supplementary Table S5). The proportion of patients ruled out by the 300 pg/mL threshold decreased substantially with age (73% in those <50 years and 8.7% in those ≥80 years) (*Figure 2B*).

**Figure 3:**
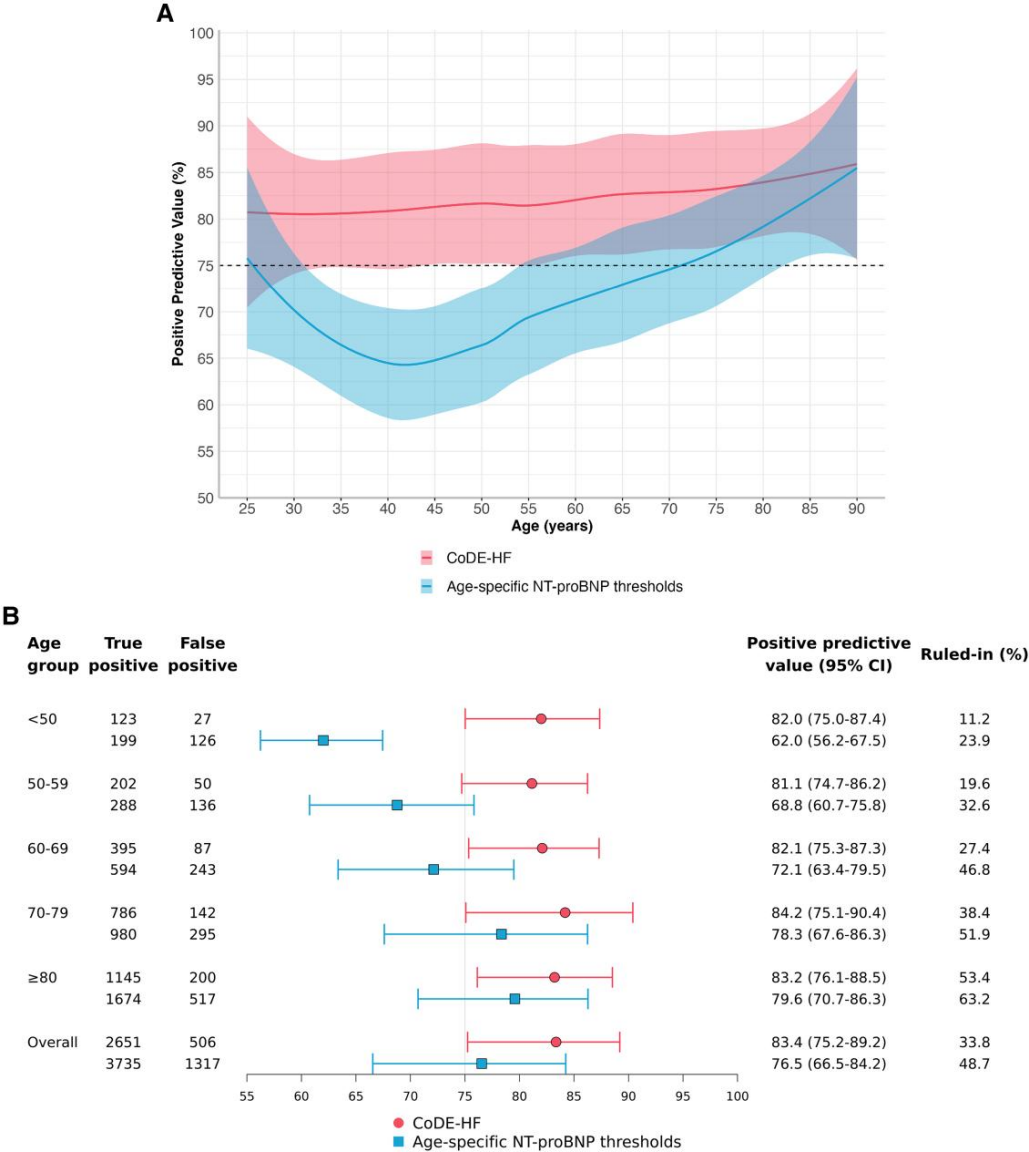
Positive predictive value of age-specific N-terminal-pro B-type natriuretic peptide rule-in thresholds and CoDE-HF high-probability score. (Age-specific NT-proBNP thresholds: 450, 900, and 1800 pg/mL for those <50 years, 50–75 years, and >75 years, respectively.) (A) Positive predictive value across age as a continuous variable. (B) Forest plot of positive predictive value across patient age groups. AUC, area under receiver operator curve; CI, confidence interval; NT-proBNP, N-terminal-pro B-type natriuretic peptide; CoDE-HF, Collaboration for the Diagnosis and Evaluation of Heart Failure

The PPV of guideline-recommended age-specific rule-in thresholds declined below 75% in patients younger than 55 years (*Figure 3A*). The PPV of these thresholds was 62.0% (56.2%–67.5%) in those <50 and 79.6% (70.7%–86.3%) in those ≥80 years (*Figure 3B*). Specificity was higher in younger patients compared with older patients [87.8% (79.8%–93%) <50 years and 68.1% (61.2%–74.3%) in those ≥80 years] (Supplementary Table S5). The proportion of patients ruled in by the age-specific thresholds was higher in older compared with younger patients (23.9% in those <50 years and 63.2% for those ≥80 years) (*Figure 3B*).

### Diagnostic performance of CoDE-HF

The discrimination of CoDE-HF was also influenced by increasing age but remained robust across all age groups [AUC of 0.971 (0.962–0.980) in those <50 and 0.881 (0.868–0.895) in those ≥80] (*Figure 1B*). CoDE-HF achieved a sensitivity of 99.2% (98.5%–99.6%) and an NPV of 98.5% (97.7%–99.1%) for the overall study population (Supplementary Table S6). The low-probability threshold of CoDE-HF achieved a consistent NPV above 98% across all age groups [NPV of 99.5% (98.8%–99.8%) in those <50 years and 98.1% (92.9%–99.5%) in those ≥80 years] (*Figure 2*). The sensitivity achieved by CoDE-HF was lower in younger patients and improved with age but remained higher than the recommended guidelines [97.7% (94.6%–99%) vs 95% (91.1%–97.2%) <50 years, respectively] (Supplementary Tables S5 and S6). The negative likelihood ratio of the CoDE-HF low-probability threshold remained lower than the NT-proBNP threshold of 300 pg/mL across all age groups (Supplementary Fig. S1). The proportion of patients ruled out decreased substantially with increasing age (71.5% in those <50 years and 4.3% for those ≥80 years) (*Figure 2B*). In an exploratory analysis, CoDE-HF achieved a higher NPV than NT-proBNP thresholds of 100, 200, and 300 pg/mL across the age continuum (Supplementary Fig. S2).

The PPV of the CoDE-HF high-probability threshold achieved a consistent PPV above 75% across all age groups [PPV of 82.0% (75.0%–87.4%) in <50 years and 83.4% (75.2%–89.2%) in those ≥80 years] (*Figure 3A*). Specificity was lower in older patients [97.9% (95.7%–99%) <50 years and 80.8% (73.8%–74.3%) ≥80 years] but was higher than the age-specific NT-proBNP thresholds across all age groups (Supplementary Tables S5 and S6). The positive likelihood ratio of the CoDE-HF high-probability threshold was similar to or higher than the age-specific NT-proBNP thresholds across all age groups (Supplementary Fig. S3). CoDE-HF ruled in fewer patients overall compared with age-specific thresholds (33.8% vs 48.7%, respectively) and the proportion of patients ruled in varied by age group, with 11.2% in those <50 years and 53.4% for those ≥80 years (*Figure 3B*). Similar results were observed in a sensitivity analysis restricted to patients with no prior diagnosis of heart failure (Supplementary Figs. S4–S6).

## Discussion

In this secondary analysis of an individual patient-level data meta-analysis,^15^ we evaluated the diagnostic performance of guideline-recommended NT-proBNP thresholds and a novel decision-support tool called CoDE-HF for the diagnosis of acute heart failure across age groups. We made several important findings which inform the use and interpretation of natriuretic peptides. First, the NPV of the guideline-recommended NT-proBNP rule-out threshold of 300 pg/mL decreases significantly with age. Beyond the age of 65 years, the NPV decreases below 98%, such that the false negative rate was over one in ten in patients older than 80 years. Second, the PPV of the age-specific NT-proBNP thresholds remained variable across age, with a significantly lower PPV in younger patients below the age of 50 compared with older patients. Finally, compared with the guideline-recommended NT-proBNP thresholds, the CoDE-HF score had a consistent and improved diagnostic performance for both the rule-in and rule-out of acute heart failure across all patient age groups.

Previous studies have been performed in relatively small and selected cohorts with a limited number of older and younger patients to fully evaluate the performance of natriuretic peptides in these important groups of patients.^26,27,33,34^ The availability of individual patient-level data in this analysis allowed a detailed evaluation of the diagnostic performance of current guideline-recommended thresholds across the age continuum. All studies included in the analysis were performed prospectively with the final diagnosis adjudicated by panels of clinicians using all available clinical information.

Natriuretic peptide concentrations are known to increase with age. Several potential mechanisms have been suggested, including an increase in diastolic dysfunction, a reduction in natriuretic peptide clearance, and a higher prevalence of comorbidities such as chronic kidney disease and atrial fibrillation with age.^35,36^ This observation has led to the development of age-specific NT-proBNP thresholds to improve the rule-in performance for acute heart failure.^33,37^ Although an important clinical advance, we observed that the diagnostic performance of age-specific thresholds continued to vary across age, with a false positive rate of two in five in those younger than 50 compared with one in five in those older than 80. We also observed a significant reduction in the diagnostic performance of the uniform NT-proBNP threshold of 300 pg/mL with age, particularly in patients older than 80 years, having a false negative rate of one in eight.

The findings of this pooled, retrospective analysis require validation and further understanding of the factors that undermine NPV and PPV in different age groups. Recent studies based on dichotomous cut-offs have shown consistent performance of age-stratified cut-offs.^38,39^ Age-related differences in heart failure phenotypes may help explain the variation in diagnostic performance across age groups. For instance, reduced sensitivity in older individuals may be influenced by a higher prevalence of heart failure with preserved ejection fraction (HFpEF), which can present with less typical biomarker profiles. Conversely, reduced specificity in younger individuals may reflect a higher prevalence of subclinical structural heart disease, often referred to as Stage B heart failure, in which NT-proBNP may be elevated despite the absence of clinical symptoms. Dichotomous thresholds are limited in detecting clinically relevant subclinical disease, such as stage B heart failure in young patients and the impact of multimorbidity in elderly individuals; thresholds alone cannot adequately take into account these factors in the interpretation of NT-proBNP, but a tool like CoDE-HF could be highly valuable.

In contrast to the NT-proBNP thresholds, we observed that CoDE-HF had a consistent and more accurate performance across age. CoDE-HF had higher discrimination than NT-proBNP in a receiver operator curve analysis and maintained a negative and PPV of greater than 98% and 75%, respectively, across all patient age groups. By incorporating age and NT-proBNP as continuous variables in the machine learning algorithm, CoDE-HF was able to optimize the diagnostic performance of NT-proBNP across age. Importantly, CoDE-HF also outperformed the age-specific NT-proBNP rule-in thresholds, suggesting that adjust for age alone is insufficient to maintain the diagnostic accuracy of NT-proBNP. CoDE-HF adjusts for other important patient factors such as renal function, body mass index and prior comorbidities that are also likely to modify the relationship between natriuretic peptide concentrations and age.^40–42^ These differences reflect not only underlying biological variation but also how guideline thresholds are applied operationally in clinical practice.

We believe our findings are important and could inform the use of natriuretic peptides in the assessment of patients with suspected acute heart failure in our ageing populations. Older patients are more likely to have other life-threatening conditions that can mimic the signs and symptoms of acute heart failure. They are also more likely to have comorbidities that influence natriuretic peptide concentrations, contributing to diagnostic uncertainty. Indeed, in our study, patients older than 80 had over four times higher prevalence of previous heart failure and ischaemic heart disease and approximately ten times higher prevalence of atrial fibrillation and chronic kidney disease. Although age-stratification in part adjusts for the accumulation of comorbidities that affect the diagnostic performance of NT-proBNP, approaches that specifically consider both age and comorbidities are likely to outperform current rule-out strategy that disregards age or adjustment for age alone for the rule-in of acute heart failure. This is consistent with previous studies demonstrating the predictive value of comorbidities in assessing the health status of individuals beyond chronological age alone.^43,44^ Whilst CoDE-HF rules in fewer patients than the age-specific thresholds, we believe this is a strength of this algorithm since a higher PPV of the high-probability score would allow clinicians to select those most likely to benefit from early specialist investigations and evidence-based therapies.^4^

The extensive external validation of our tool provides confidence in the clinical performance of the model but to encourage clinical implementation, there is a plan to evaluate its real-world impact in a randomized trial, work with multidisciplinary clinicians to integrate the tool within existing workflows and electronic healthcare record, and promote AI literacy among clinicians. Recent studies have explored other machine-learning approaches for acute heart failure diagnosis and prognosis, highlighting the growing role of data-driven decision support in this field.^45–47^ While CoDE-HF has the potential to enhance diagnostic accuracy and improve efficiency in the emergency department, its integration into routine clinical workflows presents practical challenges. Differences in electronic health record compatibility, clinician adoption, and the need for real-time data availability may influence implementation. Future studies should focus on prospective validation in diverse emergency settings to evaluate its impact on clinical decision-making, patient outcomes, and resource utilization. Additionally, demonstrating its superiority over existing NT-proBNP-based approaches in large, multicentre trials will be crucial for gaining guideline endorsement and supporting widespread adoption in clinical practice.

There are several limitations to our analysis. First, the adjudicated diagnosis of acute heart failure did not differentiate between heart failure with reduced and preserved ejection fraction. Heart failure with preserved ejection fraction is more prevalent in older patients, and NT-proBNP is known to be lower in HFpEF, which might explain the lower NPV seen in older study participants.^48^ Nevertheless, current guidelines on NT-proBNP thresholds do not differentiate between those with preserved or reduced ejection fraction.^9–14^ Second, acute heart failure is a clinical syndrome, and there will be some inherent uncertainty and variability in the diagnostic adjudication across studies, particularly in older patients. Importantly, biomarkers and decision-support tools such as CoDE-HF should be interpreted as complementary aids to, rather than substitutes for, clinician judgment. Finally, this is a retrospective evaluation of the diagnostic performance of CoDE-HF in studies where it was not used to guide clinical decisions.

## Conclusions

The diagnostic performance of guideline-recommended uniform and age-specific NT-proBNP thresholds for acute heart failure varies significantly with patient age. A machine learning decision-support tool called CoDE-HF that incorporates NT-proBNP with age as continuous variables along with comorbidities and important clinical measurements had a more consistent and accurate diagnostic performance across age groups.

## Author contributions

Conceptualization: K.K.L., D.D., N.L.M.; Investigation (Data acquisition): K.K.L., C.C.-G., C.d.F., G.M., J.H.W.R., L.G., M.M., M.Be., M.Bo., P.N., P.B., T.M., A.M.R., C.M., J.L.J.; Formal analysis: D.P.V., D.D.; Data interpretation (Validation): K.K.L., D.D., D.P.V., A.J.F.T., N.L.M.; Writing—original draft: K.K.L., D.P.V., D.D.; Writing—review and editing: All authors; Guarantors: K.K.L., D.P.V., D.D., N.L.M.

## Supplementary Material

xvaf006_Supplementary_Data

## Declarations

### Data Availability

The R code and anonymized data used to develop and validate the CoDE-HF score can be made available to researchers on request to the corresponding author.
