# Supplementary material for: Machine learning to optimize the diagnostic performance of natriuretic peptides for acute heart failure across age groups
Source: ESC Heart Fail. 2026 Jan 8;13(1):xvaf006. doi: 10.1093/eschf/xvaf006 (PMC13108261; doi:10.1093/eschf/xvaf006)

SUPPLEMENTAL MATERIAL

**Machine learning to optimise the diagnostic performance**

**of natriuretic peptides for acute heart failure across age groups**

Daniel Perez Vicencio, MSc^1,2^*, Dimitrios Doudesis, PhD^1, 2^*,
Alexander JF Thurston, BMBCh ^1^, Camille Chenevier-Gobeaux, PharmD PhD^3^,

Yann-Erick Claessens, MD^4^, Pedro Lopez-Ayala, MD^5^, Maria Belkin, MD^5^,

Desiree Wussler, MD^5, 6^, Christopher deFilippi, MD^7^, Stephen Seliger, MD^8^,

Gordon Moe, MD^9^, Carlos Fernando, MD^9^, Antoni Bayes-Genis, MD^10^,

Yigal Pinto, MD^11^, Hanna K Gaggin, MD^12, 13^, Jan C Wiemer, PhD^14^,

Martin Möckel, MD^15^, Joost HW Rutten, MD^16^, Luna Gargani, MD^17^,

Nicola R Pugliese, MD^18^ Christopher Pemberton, PhD^19^,

Irwani Ibrahim, MD^20^, Alfons Gegenhuber, MD^21^, Thomas Mueller, MD^22^,

Michael Neumaier, MD^23^, Michael Behnes, MD^24^ Ibrahim Akin, MD^24^,

Michele Bombelli, MD^25^, Guido Grassi, MD^25^,

Peiman Nazerian, MD^26^, Giovanni Albano, MD^26^,

Philipp Bahrmann, MD^27^, A Mark Richards, MD^19, 28^,

John JV McMurray, MD^29^, Christian Mueller, MD^5^,

James L Januzzi, MD^12, 13^, Nicholas L Mills, MD^1, 2^,

Kuan Ken Lee, MD^1^

*on behalf of the CoDE-HF investigators*

*Contributed equally

^1^ British Heart Foundation (BHF) Centre for Cardiovascular Science, University of Edinburgh, Edinburgh, UK.

^2^ Usher Institute, University of Edinburgh, Edinburgh, UK.

^3^ Department of Biochemistry, Cochin Hospital, Assistance Publique-Hopitaux de Paris, Paris, France.

^4^ Department of Emergency Medicine, Princess Grace Hospital Center, Monaco, Principalty of Monaco.

^5^ Cardiovascular Research Institute of Basel, Department of Cardiology, University Hospital Basel, Basel, Switzerland.

^6^ Department of Cardiology, Vancouver General Hospital, University of British Columbia, Canada.

^7^ Division of Cardiology, University of Maryland School of Medicine, Baltimore, USA.

^8^ Division of Nephrology, University of Maryland School of Medicine, Baltimore, USA.

^9^ University of Toronto, St Michael’s Hospital, Toronto, Ontario, Canada.

^10^ Heart Institute, Hospital Universitari Germans Trias i Pujol, Badalona, CIBERCV, Spain.

^11^ University of Amsterdam, Amsterdam, The Netherlands.

^12^ Harvard Medical School, Boston, Massachusetts, USA.

^13^ Division of Cardiology, Massachusetts General Hospital, Boston, Massachusetts, USA.

^14^ BRAHMS, Thermo Fisher Scientific, Hennigsdorf, Germany.

^15^ Department of Emergency and Acute Medicine with Chest Pain Units, Charité – Universitätsmedizin Berlin, Campus Mitte and Virchow, Berlin, Germany.

^16^ Department of Internal Medicine, Radboud University Medical Center, Nijmegen, The Netherlands.

^17^ Department of Surgical, Medical and Molecular Pathology and Critical Care Medicine, University of Pisa, Pisa, Italy.

^18^ Department of Clinical and Experimental Medicine, University of Pisa, Pisa, Italy.

^19^ Christchurch Heart Institute, University of Otago, Christchurch, New Zealand.

^20^ Emergency Medicine Department, National University Hospital, Singapore.

^21^ Department of Internal Medicine, Krankenhaus Bad Ischl, Bad Ischl, Austria.

^22^ Department of Laboratory Medicine, Hospital Voecklabruck, Austria.

^23^ Institute for Clinical Chemistry, University Medical Centre Mannheim, Faculty of Medicine Mannheim, University of Heidelberg, Mannheim, Germany.

^24^ First Department of Medicine, University Medical Centre Mannheim, Faculty of Medicine Mannheim, University of Heidelberg, Mannheim, Germany.

^25^ Clinica Medica, University of Milano – Bicocca, Monza, Italy.

^26^ Department of Emergency Medicine, Azienda Ospedaliero-Universitaria Careggi, Florence, Italy.

^27^ Institute for Biomedicine of Aging, Friedrich-Alexander-University, Nuremberg, Germany.

^28^ Cardiovascular Research Institute, National University Heart Centre Singapore, Singapore.

^29^ BHF Cardiovascular Research Centre, University of Glasgow, Glasgow, UK.

**Corresponding Author:**

Dr Kuan Ken Lee

BHF/University Centre for Cardiovascular Science

The University of Edinburgh

Edinburgh EH16 4SA

United Kingdom

Telephone: 0044 131 242 6515

E-mail: [ken.lee@ed.ac.uk](mailto:ken.lee@ed.ac.uk)

**Supplemental Tables: 5**

**Supplemental Figures: 6**

| **Supplementary Table 1**. Characteristics of each study included. | | | | | |
| --- | --- | --- | --- | --- | --- |
| **Author, year** | **NT-proBNP assay** | **Study design** | **Country** | **Cohort size** | **Setting** |
| Bahrmann et al, 2015^7^ | Roche | Prospective cohort | Germany | 303 | Emergency department |
| Behnes et al, 2009^8^ | Dade Behring | Prospective cohort | Germany | 401 | Emergency department |
| Bombelli et al, 2015^9^ | Roche | Prospective cohort | Italy | 895 | Emergency department |
| Chenevier-Gobeaux et al, 2005^10^ | Roche | Prospective cohort | France | 380 | Emergency department |
| deFilippi et al, 2007^11^ | Roche | Prospective cohort | USA | 831 | Emergency department |
| Gargani et al, 2008^12^ | Roche | Prospective cohort | Italy | 149 | Cardiology and pulmonary hospital admissions |
| Ibrahim et al, 2017^13^ | Roche | Prospective cohort | Singapore, New Zealand | 1106 | Emergency department |
| Januzzi et al, 2006^14^ | Roche | Prospective cohort | New Zealand, Spain, USA | 1256 | Emergency department |
| Maisel et al, 2010^15^ | Roche | Prospective cohort | USA, Germany, Switzerland, Italy, Greece, UK, Poland, New Zealand | 1623 | Emergency department |
| Moe et al, 2007^16^ | Roche | Randomized controlled trial | Canada | 500 | Emergency department |
| Mueller et al, 2005^17^ | Roche | Prospective cohort | Austria | 251 | Emergency department |
| Nazerian et al, 2010^18^ | Roche | Prospective cohort | Italy | 145 | Emergency department |
| Rutten et al, 2008^19^ | Roche | Randomized controlled trial | The Netherlands | 476 | Emergency department |
| Wussler et al, 2019^20^ | Roche | Prospective cohort | Switzerland | 2053 | Emergency department |

| **Author, year** | **Inclusion criteria** | **Exclusion criteria** |
| --- | --- | --- |
| Bahrmann et al, 2015^7^ | All consecutive non-trauma patients aged ≥70 years who were admitted to the Emergency Department. | Patients with acute ST-elevation myocardial infarction, planned elective coronary revascularisation, hospitalization for unstable angina within the preceding 2 months, coronary-artery bypass grafting or percutaneous transluminal angioplasty within the preceding 3 months. Patients were also excluded if they had renal failure requiring dialysis, trauma with suspected myocardial contusion, life expectancy <6 months, or if they did not consent to providing a blood sample for use by the research team. |
| Behnes et al, 2009^8^ | Consecutive patients presenting with symptoms of acute dyspnoea and/ or peripheral oedema in the Emergency Department. | Patients suffering from severe renal disease (defined as serum creatinine level greater than 2.8 mg/dl) or anemia (hemoglobin concentrations below 8.0 g/dl) were excluded. Further exclusion criteria were obvious traumatic causes of dyspnea, pregnancy, a status after immediate cardiopulmonary resuscitation, participation in another clinical trial and age under 18 years. |
| Bombelli et al, 2015^9^ | Consecutive patients aged 80 years or more evaluated in the Emergency Department in whom NT-proBNP was measured. |  |
| Chenevier-Gobeaux et al, 2005^10^ | Consecutive patients presenting to the Emergency Department with dyspnoea. |  |
| deFilippi et al, 2007^11^ | Consecutive patients presenting to the Emergency Department with dyspnoea and who underwent measurement of a natriuretic peptide at presentation. | Patients younger than 18 years or in whom there was inadequate clinical information recorded to assess the aetiology of dyspnoea were excluded. |
| Gargani et al, 2008^12^ | Patients with dyspnoea at admission as reported on the case history, had a venous blood sample taken for NT-proBNP analysis on the day of admission, underwent assessment for ultrasound lung comets performed within 4 h of the NT-proBNP measurement and did not receive diuretic therapy between the two measurements. |  |
| Ibrahim et al, 2017^13^ | Shortness of breath as the primary complaint triggering presentation to the Emergency Department. | Age under 21 years, shortness of breath related to trauma, and current renal haemodialysis were exclusion criteria. |
| Januzzi et al, 2006^14^ | Dyspnoeic Emergency Department patients. |  |
| Maisel et al, 2010^15^ | Patients reporting shortness of breath as primary complaint in the Emergency Department. | Patients <18 years of age, unable to provide consent, had an acute ST-segment elevation myocardial infarction, were receiving haemodialysis, or had renal failure. |
| Moe et al, 2007^16^ | Consecutive patients >18 years of age presenting to the Emergency Department with dyspnoea of suspected cardiac origin. | Patients with advanced renal failure (serum creatinine >250 micromol/L), acute myocardial infarction, malignant disorders, and dyspnoea from clinically overt origins, including pneumothorax and chest wall trauma. |
| Mueller et al, 2005^17^ | Consecutive patients presenting with dyspnoea as chief complaint to the Emergency Department. | Patients with ST elevation myocardial infarction, non-ST elevation myocardial infarction, or acute coronary syndrome troponin positive and trauma patients. |
| Nazerian et al, 2010^18^ | Convenience sample of patients presenting to the Emergency Department with acute dyspnoea as the main symptom. | Patients with trauma, ST-elevation myocardial infarctions, or dyspnoea clearly caused by something other than heart failure, such as pneumothorax, were excluded. Patients were also excluded if they had received intravenous therapy in the Emergency Department before echocardiogram and NT-proBNP were performed. Patients who met the inclusion criteria were invited to participate in the study. Echocardiogram was performed in all patients who met the inclusion criteria. However, if the investigator judged that both left ventricular (LV) ejection fraction and pulsed Doppler analysis of mitral inflow were not interpretable due to a very poor acoustic window, the patient was excluded from the study because echocardiogram was not feasible. |
| Rutten et al, 2008^19^ | Patients were eligible if they presented with acute dyspnoea as their most prominent complaint. | Patients with acute dyspnoea due to trauma or cardiogenic shock and patients with renal failure requiring haemodialysis or peritoneal dialysis were excluded. |
| Wussler et al, 2019^20^ | Adult patients presenting with acute dyspnoea to the Emergency Department. | Patients with terminal kidney failure requiring haemodialysis were excluded. |

| **Author, year** | **Diagnostic adjudication for acute heart failure** | **Risk of bias (QUADAS-2)** |
| --- | --- | --- |
| Bahrmann et al, 2015^7^ | Independent adjudication by two cardiologists based on the definition of the ESC guideline. They reviewed all available medical records of the index hospital stay, including the clinical history findings from the physical examination, results of laboratory tests (excluding NT-proBNP), radiographic studies, ECG, and echocardiography. | Patient selection: high; index test: low; reference standard: low; flow and timing: low; Overall: high. |
| Behnes et al, 2009^8^ | Retrospective review by a study physician who had unrestricted access to the records of the patients but was blinded to the results of NT-proBNP measurements. | Patient selection: low; index test: low; reference standard: low; flow and timing: low; Overall: low. |
| Bombelli et al, 2015^9^ | Adjudication by clinician based on Framingham criteria. | Patient selection: high; index test: low; reference standard: low; flow and timing: low; Overall: high. |
| Chenevier-Gobeaux et al, 2005^10^ | Independent adjudication by two ED clinicians on the basis of clinical examination, medical history, ECG, chest X-ray and blood analysis (including plasma creatinine). | Patient selection: low; index test: low; reference standard: low; flow and timing: low; Overall: low. |
| deFilippi et al, 2007^11^ | Adjudication by cardiologist who reviewed case report forms blinded to natriuretic peptide results. Subset of 50 random cases adjudicated by second cardiologist, demonstrating good agreement. | Patient selection: high; index test: low; reference standard: low; flow and timing: low; Overall: high. |
| Gargani et al, 2008^12^ | Independent adjudication by two cardiologists who were blinded to the NT- proBNP values, who reviewed all of the medical records pertaining to the patient. | Patient selection: high; index test: low; reference standard: low; flow and timing: high; Overall: high. |
| Ibrahim et al, 2017^13^ | Independent adjudication by an ED specialist and a cardiologist. They were blinded to NT-proBNP measurements but could access medical records, case report forms, and other test results including cardiac imaging as available. | Patient selection: low; index test: low; reference standard: low; flow and timing: low; Overall: low. |
| Januzzi et al, 2006^14^ | Adjudicated by a panel of physicians or cardiologists utilizing all available clinical data, blinded to NTproBNP results. | Patient selection: low; index test: low; reference standard: low; flow and timing: low; Overall: low. |
| Maisel et al, 2010^15^ | Independent adjudication by two cardiologists who reviewed all medical records. | Patient selection: low; index test: low; reference standard: low; flow and timing: low; Overall: low. |
| Moe et al, 2007^16^ | Independent adjudication by two cardiologists. They were provided with hospital records, including the discharge summary, results of laboratory and radiographic testing, echocardiograms if performed, clinical notes from the time of ED presentation to the 60-day follow-up, and outcome of a telephone interview. Using all available data, the cardiologists assigned a diagnosis without knowledge of the NT- proBNP results. | Patient selection: low; index test: low; reference standard: low; flow and timing: low; Overall: low. |
| Mueller et al, 2005^17^ | Retrospective review of all medical records by a clinician based on Framingham criterion | Patient selection: low; index test: low; reference standard: low; flow and timing: low; Overall: low. |
| Nazerian et al, 2010^18^ | Independent adjudication by two cardiologists and one respiratory physician, blinded to echocardiogram and NTproBNP results. The reviewers had access to ED records, clinical notes, components and summary of the Framingham Heart Study Criteria and any additional information that became available during hospital stay. | Patient selection: high; index test: low; reference standard: low; flow and timing: low; Overall: high. |
| Rutten et al, 2008^19^ | Consensus between two clinicians in internal medicine, pulmonology or cardiology. | Patient selection: low; index test: low; reference standard: high; flow and timing: low; Overall: high. |
| Wussler et al, 2019^20^ | Adjudicated by 2 independent cardiologist-internists who had access to all patients' medical records, including clinical history, physical examination, 12-lead electrocardiograms, laboratory findings, chest radiographs, echocardiograms, lung function test results, computed tomography scans, and response to therapy, as well as autopsy data for patients who died in the hospital. | Patient selection: high; index test: low; reference standard: high; flow and timing: low; Overall: high. |
| Abbreviations: ESC= European society of cardiology; ED= emergency department; ECG= electrocardiogram. | | |

| **Supplementary Table 2**. Baseline characteristics of patients within each study. | | | | | | |  |  |
| --- | --- | --- | --- | --- | --- | --- | --- | --- |
|  | **Overall** | **Bahrmann et al** | **Behnes et al** | **Bombelli et al** | **Chenevier-Gobeaux et al** | **De Filippi et al** | | |
| **Number of participants** | 10369 | 303 | 401 | 895 | 380 | 831 | | |
| **Male sex** | 5531 (53.3) | 148 (48.8) | 205 (51.1) | 368 (41.1) | 189 (49.7) | 380 (45.7) | | |
| **Age, years** | 69.3 (16.3) | 80.4 (5.9) | 67.4 (15.6) | 85.7 (4.2) | 78.5 (12.2) | 66.3 (14.9) | | |
| < 50 | 1377 (13.3) | - | 53 (13.2) | - | 5 (1.3) | 125 (15.0) | | |
| 50-75 | 4370 (42.1) | 79 (26.1) | 211 (52.6) | - | 121 (31.8) | 443 (53.3) | | |
| >75 | 4622 (44.6) | 224 (73.9) | 137 (34.2) | 895 (100.0) | 254 (66.8) | 263 (31.6) | | |
| **Ethnicity** |  |  |  |  |  |  | | |
| Black | 845 (14.8) | 0 (0.0) | NR | NR | NR | 318 (38.3) | | |
| White | 4112 (72.1) | 303 (100.0) | NR | NR | NR | 499 (60.0) | | |
| Other | 743 (13.0) | 0 (0.0) | NR | NR | NR | 14 (1.7) | | |
| **Past medical history** |  |  |  |  |  |  | | |
| Prior Heart failure | 3119 (33.4) | 224 (73.9) | 194 (48.4) | NR | 128 (33.8) | 287 (36.1) | | |
| Ischemic heart disease | 2953 (32.3) | 138 (45.5) | 157 (39.2) | NR | 124 (32.6) | 263 (33.1) | | |
| Diabetes Mellitus | 2398 (26.7) | 117 (38.6) | 120 (30.1) | NR | NR | 305 (38.2) | | |
| Hypertension | 5071 (59.3) | 255 (84.4) | 268 (67.0) | NR | 153 (40.3) | NR | | |
| Hyperlipidemia | 2269 (41.2) | 206 (68.0) | 122 (30.7) | NR | NR | NR | | |
| Current or ex-smoker | 2458 (41.3) | 149 (49.2) | 206 (53.9) | NR | NR | NR | | |
| Asthma | 770 (18.5) | NR | 21 (5.2) | NR | NR | NR | | |
| COPD | 2117 (29.2) | 87 (28.7) | 94 (23.4) | NR | 127 (33.4) | NR | | |
| Atrial fibrillation | 1701 (20.9) | 89 (29.4) | 70 (17.5) | NR | NR | 175 (22.0) | | |
| Chronic Kidney Disease | 1215 (18.9) | 18 (5.9) | 71 (17.7) | NR | NR | NR | | |
| **Body mass index, kg/m^2^** | 27.7 (7.2) | 27.1 (5.0) | 27.9 (6.2) | NR | NR | 30.3 (9.7) | | |
| <25 | 3062 (39.0) | 104 (34.3) | 118 (32.6) | NR | NR | 195 (31.5) | | |
| 25-30 | 2473 (31.5) | 121 (39.9) | 146 (40.3) | NR | NR | 172 (27.8) | | |
| ≥30 | 2317 (29.5) | 78 (25.7) | 98 (27.1) | NR | NR | 252 (40.7) | | |
| **Physiological parameters** |  |  |  |  |  |  | | |
| Heart rate, beats per minute | 91.7 (23.7) | 85.2 (23.2) | 91.7 (24.0) | NR | NR | NR | | |
| Systolic blood pressure, mmHg | 140.0 (27.9) | 146.5 (27.3) | 139.2 (28.6) | NR | NR | NR | | |
| Diastolic blood pressure, mmHg | 79.7 (17.0) | 74.9 (15.7) | 79.0 (13.7) | NR | NR | NR | | |
| **Clinical hematology and biochemistry** |  |  |  |  |  |  | | |
| Hemoglobin, g/dL | 13.1 (2.1) | 12.4 (2.3) | 13.2 (1.9) | NR | NR | NR | | |
| eGFR, mL/min/1.73m^2^ | 68.2 (31.3) | 48.0 (14.8) | 66.8 (24.9) | 52.9 (25.4) | 54.7 (19.9) | 63.3 (34.0) | | |
| NT proBNP, pg/mL | 1182.2 [191.0, 4737.0] | 1594.0 [433.5, 4976.5] | 766.0 [150.6, 3155.1] | 2937.0 [1014.0, 7598.5] | 1678.0 [409.8, 5875.0] | 1731.0 [472.0, 6032.5] | | |
| **Adjudicated diagnosis of heart failure** | 4549 (43.9) | 168 (55.4) | 122 (30.4) | 405 (45.3) | 115 (30.3) | 437 (52.6) | | |
| Presented as No. (%), mean (SD) or median [inter-quartile range]. Abbreviations: COPD= chronic obstructive pulmonary disease; eGFR= estimated glomerular filtration rate; NT-proBNP= N-terminal pro-B-type natriuretic peptide; CVD= cardiovascular disease; NR= not reported. | | | | | | | |  |

|  | Gargani et al | Ibrahim et al | Januzzi et al | Maisel et al | Moe et al | Mueller et al |
| --- | --- | --- | --- | --- | --- | --- |
| Number of participants | 149 | 1106 | 1256 | 1623 | 500 | 251 |
| Male sex | 98 (65.8) | 683 (61.8) | 643 (51.2) | 848 (52.2) | 258 (51.6) | 234 (93.2) |
| Age, years | 70.8 (11.0) | 62.1 (16.2) | 68.4 (15.9) | 63.8 (16.9) | 70.7 (14.3) | 70.2 (14.0) |
| < 50 | 5 (3.4) | 232 (21.0) | 183 (14.6) | 332 (20.5) | 46 (9.2) | 24 (9.6) |
| 50-75 | 91 (61.1) | 601 (54.3) | 554 (44.1) | 800 (49.3) | 231 (46.2) | 115 (45.8) |
| >75 | 53 (35.6) | 273 (24.7) | 519 (41.3) | 491 (30.3) | 223 (44.6) | 112 (44.6) |
| Ethnicity |  |  |  |  |  |  |
| Black | 0 (0.0) | 0 (0.0) | 46 (3.7) | 471 (29.3) | 10 (2.0) | 0 (0.0) |
| White | 149 (100.0) | 461 (41.7) | 1210 (96.3) | 1078 (67.0) | 464 (93.0) | 251 (100.0) |
| Other | 0 (0.0) | 645 (58.3) | 0 (0.0) | 59 (3.7) | 25 (5.0) | 0 (0.0) |
| Past medical history |  |  |  |  |  |  |
| Prior Heart failure | 46 (30.9) | 235 (21.3) | 429 (34.2) | 563 (35.6) | 171 (38.2) | 75 (29.9) |
| Ischemic heart disease | 60 (40.3) | 110 (11.8) | 526 (42.0) | 498 (31.7) | 135 (31.1) | 117 (46.6) |
| Diabetes Mellitus | 57 (38.3) | 293 (26.5) | 309 (24.6) | 457 (28.5) | 113 (26.2) | 58 (23.1) |
| Hypertension | 79 (53.0) | 601 (54.3) | 661 (52.6) | 1069 (66.9) | 262 (58.9) | 141 (56.2) |
| Hyperlipidemia | 57 (38.3) | 472 (43.1) | NR | NR | NR | NR |
| Current or ex-smoker | 13 (9.6) | 245 (22.2) | 721 (57.5) | 463 (29.4) | 311 (65.8) | 46 (100.0) |
| Asthma | 2 (1.3) | 282 (25.6) | NR | 313 (19.9) | 88 (19.6) | NR |
| COPD | 33 (22.1) | 254 (23.0) | NR | 465 (29.5) | 126 (30.3) | 72 (28.7) |
| Atrial fibrillation | 30 (27.0) | 164 (16.1) | 280 (22.3) | 240 (16.8) | NR | 83 (33.1) |
| Chronic Kidney Disease | 43 (29.1) | 121 (11.0) | NR | 244 (15.6) | NR | 74 (29.5) |
| Body mass index, kg/m^2^ | 27.7 (5.1) | 27.4 (7.5) | 27.4 (6.3) | 29.2 (8.8) | 28.5 (7.4) | 26.8 (5.0) |
| <25 | 42 (30.7) | 308 (43.0) | 412 (37.4) | 490 (35.4) | 157 (33.0) | 93 (37.1) |
| 25-30 | 54 (39.4) | 195 (27.2) | 375 (34.0) | 384 (27.7) | 162 (34.0) | 100 (39.8) |
| ≥30 | 41 (29.9) | 213 (29.7) | 316 (28.6) | 511 (36.9) | 157 (33.0) | 58 (23.1) |
| Physiological parameters |  |  |  |  |  |  |
| Heart rate, beats per minute | 78.1 (16.6) | 93.2 (23.0) | 91.5 (24.9) | 91.4 (22.8) | 86.5 (21.7) | 93.8 (26.1) |
| Systolic blood pressure, mmHg | 131.7 (23.4) | 139.9 (26.9) | NR | 140.9 (28.7) | 136.1 (25.4) | 140.1 (31.9) |
| Diastolic blood pressure, mmHg | 73.1 (10.8) | 79.9 (16.1) | NR | 80.8 (17.4) | 76.8 (17.0) | 81.8 (16.3) |
| Clinical hematology and biochemistry |  |  |  |  |  |  |
| Hemoglobin, g/dL | 13.4 (0.4) | 13.4 (2.0) | 13.0 (2.0) | 12.9 (2.2) | 13.0 (1.8) | 13.7 (2.0) |
| eGFR, mL/min/1.73m^2^ | 59.9 (23.1) | 72.9 (27.7) | 72.5 (31.9) | 70.4 (30.4) | 76.4 (30.1) | 94.0 (44.6) |
| NT proBNP, pg/mL | 2303.0 [430.0, 5034.0] | 531.8 [87.7, 3149.1] | 1403.5 [150.1, 6284.5] | 832.9 [111.5, 4114.5] | 1293.5 [212.8, 4199.5] | 1222.0 [269.4, 3981.5] |
| Adjudicated diagnosis of heart failure | 122 (81.9) | 327 (29.6) | 720 (57.3) | 562 (34.6) | 230 (46.0) | 137 (54.6) |

|  | Nazerian et al | Rutten et al | Wussler et al |
| --- | --- | --- | --- |
| Number of participants | 145 | 476 | 2053 |
| Male sex | 74 (51.0) | 257 (54.0) | 1146 (55.8) |
| Age, years | 77.8 (11.2) | 58.6 (17.8) | 70.5 (15.5) |
| < 50 | 4 (2.8) | 144 (30.3) | 224 (10.9) |
| 50-75 | 43 (29.7) | 239 (50.2) | 842 (41.0) |
| >75 | 98 (67.6) | 93 (19.5) | 987 (48.1) |
| Ethnicity |  |  |  |
| Black | NR | NR | NR |
| White | NR | NR | NR |
| Other | NR | NR | NR |
| Past medical history |  |  |  |
| Prior Heart failure | 30 (20.7) | 85 (17.9) | 652 (32.0) |
| Ischemic heart disease | 47 (32.4) | 100 (21.0) | 678 (33.2) |
| Diabetes Mellitus | 25 (17.2) | 77 (16.2) | 467 (22.8) |
| Hypertension | 84 (57.9) | 120 (25.2) | 1378 (67.5) |
| Hyperlipidemia | NR | NR | 846 (41.9) |
| Current or ex-smoker | NR | 304 (64.5) | NR |
| Asthma | NR | 64 (13.4) | NR |
| COPD | 49 (33.8) | 126 (26.5) | 684 (33.4) |
| Atrial fibrillation | 50 (34.5) | 70 (19.2) | 450 (21.9) |
| Chronic Kidney Disease | 15 (10.3) | 41 (8.6) | 588 (28.7) |
| Body mass index, kg/m^2^ | NR | 25.6 (5.7) | 26.6 (6.1) |
| <25 | NR | 247 (52.2) | 896 (44.2) |
| 25-30 | NR | 138 (29.2) | 626 (30.9) |
| ≥30 | NR | 88 (18.6) | 505 (24.9) |
| Physiological parameters |  |  |  |
| Heart rate, beats per minute | 98.3 (21.2) | 97.4 (23.9) | 92.6 (23.9) |
| Systolic blood pressure, mmHg | 137.6 (28.2) | 147.9 (33.2) | 138.5 (26.1) |
| Diastolic blood pressure, mmHg | 76.0 (15.8) | 81.5 (19.5) | 80.2 (17.4) |
| Clinical hematology and biochemistry |  |  |  |
| Hemoglobin, g/dL | 13.5 (2.3) | 13.6 (2.3) | 13.1 (2.1) |
| eGFR, mL/min/1.73m^2^ | 52.6 (22.1) | 88.7 (40.2) | 67.4 (29.2) |
| NT proBNP, pg/mL | 2355.0 [764.0, 7806.0] | 48.8 [8.3, 314.2] | 1333.0 [230.0, 5178.0] |
| Adjudicated diagnosis of heart failure | 64 (44.1) | 97 (20.4) | 1043 (50.8) |

| **Supplementary Table 3**. Baseline characteristics of study patients stratified by prior history of heart failure. | | | |  |
| --- | --- | --- | --- | --- |
|  | **Overall** | **No prior history of heart failure** | **Prior history**  **of heart failure** | |
| **Number of participants** | 10369 | 6208 | 3119 | |
| **Male sex** | 5531 (53.3) | 3271 (52.7) | 1818 (58.3) | |
| **Age, years** | 69.3 (16.3) | 64.7 (16.9) | 73.7 (12.7) | |
| < 50 | 1377 (13.3) | 1191 (19.2) | 168 (5.4) | |
| 50-75 | 4370 (42.1) | 3012 (48.5) | 1298 (41.6) | |
| >75 | 4622 (44.6) | 2005 (32.3) | 1653 (53.0) | |
| **Ethnicity** |  |  |  | |
| Black | 845 (14.8) | 522 (13.9) | 303 (16.8) | |
| White | 4112 (72.1) | 2634 (70.0) | 1373 (76.2) | |
| Other | 743 (13.0) | 609 (16.2) | 127 (7.0) | |
| **Past medical history** |  |  |  | |
| Ischemic heart disease | 2953 (32.3) | 1228 (20.4) | 1687 (55.6) | |
| Diabetes Mellitus | 2398 (26.7) | 1270 (21.5) | 1096 (37.1) | |
| Hypertension | 5071 (59.3) | 2950 (52.2) | 2057 (73.4) | |
| Hyperlipidemia | 2269 (41.2) | 1156 (32.1) | 1092 (58.5) | |
| Current or ex-smoker | 2458 (41.3) | 1645 (42.3) | 767 (38.9) | |
| Asthma | 770 (18.5) | 621 (22.1) | 131 (10.3) | |
| COPD | 2117 (29.2) | 1361 (28.3) | 718 (30.4) | |
| Atrial fibrillation | 1701 (20.9) | 822 (15.5) | 861 (31.1) | |
| Chronic Kidney Disease | 1215 (18.9) | 493 (11.4) | 712 (34.2) | |
| **Body mass index, kg/m^2^** | 27.7 (7.2) | 27.5 (7.2) | 28.0 (7.2) | |
| <25 | 3062 (39.0) | 2053 (40.6) | 963 (36.1) | |
| 25-30 | 2473 (31.5) | 1532 (30.3) | 900 (33.8) | |
| ≥30 | 2317 (29.5) | 1477 (29.2) | 801 (30.1) | |
| **Physiological parameters** |  |  |  | |
| Heart rate, beats per minute | 91.7 (23.7) | 93.2 (23.5) | 88.8 (23.9) | |
| Systolic blood pressure, mmHg | 140.0 (27.9) | 141.9 (27.1) | 136.2 (29.2) | |
| Diastolic blood pressure, mmHg | 79.7 (17.0) | 81.0 (16.7) | 77.1 (17.2) | |
| **Clinical hematology and biochemistry** |  |  |  | |
| Hemoglobin, g/dL | 13.1 (2.1) | 13.4 (2.1) | 12.6 (2.1) | |
| eGFR, mL/min/1.73m^2^ | 68.2 (31.3) | 77.0 (31.5) | 55.1 (25.8) | |
| NT proBNP, pg/mL | 1182.2 [191.0, 4737.0] | 421.5 [83.8, 2270.2] | 3484.0 [1162.5, 8905.5] | |
| **Adjudicated diagnosis of heart failure** | 4549 (43.9) | 1802 (29.0) | 2286 (73.3) | |
| Presented as No. (%), mean (SD) or median [inter-quartile range]. Abbreviations: COPD= chronic obstructive pulmonary disease; eGFR= estimated glomerular filtration rate; NT-proBNP= N-terminal pro-B-type natriuretic peptide; CVD= cardiovascular disease. | | | |  |

| **Supplementary Table 4. Baseline characteristics of patients without previous heart failure stratified by age groups.** | | | | | | |
| --- | --- | --- | --- | --- | --- | --- |
|  | **All participants**  (n = 6,208) | **<50**  (n = 1,176) | **50-59**  (n = 1,005) | **60-69**  (n = 1,210) | **70-79**  (n = 1,497) | **≥80**  (n = 1,320) |
| **Male sex** | 3,271 (53) | 572 (49) | 571 (57) | 723 (60) | 818 (55) | 587 (44) |
| **Ethnicity** |  |  |  |  |  |  |
| Black | 522 (14) | 202 (26) | 135 (20) | 93 (12) | 57 (6.7) | 35 (5.3) |
| White | 2,634 (70) | 368 (47) | 373 (54) | 570 (73) | 715 (84) | 608 (91) |
| Other | 609 (16) | 208 (27) | 180 (26) | 118 (15) | 80 (9.4) | 23 (3.5) |
| **Past medical history** |  |  |  |  |  |  |
| Ischaemic heart disease | 1,228 (20) | 49 (4.2) | 136 (14) | 242 (21) | 417 (29) | 384 (30) |
| Diabetes | 1,270 (22) | 129 (11) | 223 (23) | 316 (27) | 376 (26) | 226 (19) |
| Hypertension | 2,950 (52) | 213 (20) | 442 (49) | 595 (55) | 880 (64) | 820 (68) |
| Hyperlipidemia | 1,156 (32) | 84 (12) | 190 (31) | 283 (40) | 381 (45) | 218 (31) |
| Current smoker or ex-smoker | 1,645 (42) | 411 (47) | 343 (49) | 348 (45) | 370 (41) | 173 (27) |
| Asthma | 621 (22) | 252 (37) | 140 (26) | 102 (17) | 76 (13) | 51 (13) |
| Chronic obstructive pulmonary disease | 1,361 (28) | 94 (10) | 210 (27) | 311 (34) | 434 (38) | 312 (30) |
| Atrial fibrillation | 822 (16) | 33 (3.6) | 71 (8.2) | 130 (12) | 273 (21) | 315 (28) |
| Chronic kidney disease | 493 (11) | 11 (1.2) | 33 (4.6) | 77 (9.1) | 134 (13) | 238 (28) |
| **Body mass index, kg/m^2^** | 28 (7) | 28 (9) | 29 (8) | 28 (7) | 27 (6) | 25 (5) |
| <25 | 2,053 (41) | 431 (43) | 273 (34) | 360 (36) | 462 (37) | 527 (53) |
| 25-29 | 1,532 (30) | 243 (24) | 240 (30) | 306 (31) | 430 (35) | 313 (31) |
| ≥30 | 1,477 (29) | 332 (33) | 300 (37) | 337 (34) | 351 (28) | 157 (16) |
| **Physiological parameters** |  |  |  |  |  |  |
| Heart rate, beats per minute | 93 (23) | 95 (21) | 94 (23) | 94 (25) | 91 (25) | 92 (23) |
| Systolic blood pressure, mmHg | 142 (27) | 134 (24) | 141 (27) | 143 (28) | 144 (27) | 147 (28) |
| Diastolic blood pressure, mmHg | 81 (17) | 82 (17) | 85 (16) | 82 (17) | 79 (16) | 79 (17) |
| **Clinical hematology and biochemistry** |  |  |  |  |  |  |
| Hemoglobin, g/dL | 13.38 (2.07) | 13.86 (2.07) | 13.71 (2.07) | 13.55 (2.00) | 13.21 (2.04) | 12.73 (1.99) |
| eGFR, mL/min/1.73m^2^ | 77 (32) | 103 (30) | 88 (27) | 78 (30) | 67 (26) | 58 (23) |
| NT proBNP, pg/mL | 422 [84 - 2,270] | 49 [16 - 179] | 131 [45 - 816] | 360 [103 - 1,715] | 790 [228 - 2,803] | 2,245 [652 - 6,787] |
| **Adjudicated diagnosis of heart failure** | 1,802 (29) | 98 (8.3) | 181 (18) | 299 (25) | 550 (37) | 674 (51) |
| Values are median (interquartile range), n (%) or mean ± SD. eGFR=estimated glomerular filtration rate; NT-proBNP=N-terminal pro-B-type natriuretic peptide. | | | | | | |

| **Supplementary Table 5.** A) Diagnostic performance of NT-proBNP rule-out threshold of 300 pg/mL | | | | | | | | |  |  |
| --- | --- | --- | --- | --- | --- | --- | --- | --- | --- | --- |
| **Age group** | **True positive** | **False positive** | **True negative** | **False negative** | **Negative predictive value**  **(95% CI)** | **Sensitivity**  **(95% CI)** | **LR-**  **(95% CI)** | **Proportion ruled out**  **(%)** | | |
| <50 | 207 | 160 | 982 | 11 | 98.9  (97.6-99.5) | 95.0  (91.1-97.2) | 0.039  (0.015-0.103) | 73.0 | | |
| 50 - 59 | 343 | 279 | 655 | 23 | 98.0  (93.5-99.4) | 96.1  (88.5-98.7) | 0.025  (0.007-0.088) | 52.2 | | |
| 60 - 69 | 660 | 484 | 600 | 43 | 93.3  (89.6-95.8) | 93.8  (89.5-96.5) | 0.092  (0.048-0.174) | 36.0 | | |
| 70 - 79 | 1185 | 738 | 484 | 50 | 90.9  (85.8-94.3) | 95.8  (92.2-97.8) | 0.117  (0.064-0.215) | 21.7 | | |
| ≥80 | 1993 | 1172 | 266 | 34 | 88.7  (84.2-92.1) | 98.4  (97.2-99.0) | 0.051  (0.023-0.111) | 8.7 | | |
| Overall | 4388 | 2833 | 2987 | 161 | 94.6  (91.9-96.4) | 96.8  (94.6-98.1) | 0.075  (0.048-0.119) | 30.4 | | |
| CI: confidence interval; LR-: negative likelihood ratio. | | | | | | | | | |  |

| B) Diagnostic performance of NT-proBNP age-specific rule-in thresholds | | | | | | | | |  |
| --- | --- | --- | --- | --- | --- | --- | --- | --- | --- |
| **Age group** | **True positive** | **False positive** | **True negative** | **False negative** | **Positive predictive value**  **(95% CI)** | **Specificity**  **(95% CI)** | **LR+**  **(95% CI)** | **Proportion ruled in**  **(%)** | |
| <50 | 199 | 126 | 1016 | 19 | 62.0  (56.2-67.5) | 87.8  (79.8-93.0) | 8.9  (5.5-14.4) | 23.9 | |
| 50 - 59 | 288 | 136 | 798 | 78 | 68.8  (60.7-75.8) | 86.0  (76.9-92.0) | 5.1  (3.1-8.3) | 32.6 | |
| 60 - 69 | 594 | 243 | 841 | 109 | 72.1  (63.4-79.5) | 80.2  (72.7-86.0) | 3.8  (3.0-4.9) | 46.8 | |
| 70 - 79 | 980 | 295 | 927 | 255 | 78.3  (67.6-86.3) | 79.3  (70.4-86.1) | 3.6  (2.7-4.9) | 51.9 | |
| ≥80 | 1674 | 517 | 921 | 353 | 79.6  (70.7-86.3) | 68.1  (61.2-74.3) | 2.3  (1.8-2.9) | 63.2 | |
| Overall | 3735 | 1317 | 4503 | 814 | 76.5  (66.5-84.2) | 79.6  (71.4-86.0) | 3.8  (2.9-5.1) | 48.7 | |
| Age-specific rule-in thresholds (450, 900 and 1,800 pg/mL for patients aged <50, 50-75 and >75 years, respectively). CI: confidence interval; LR+: positive likelihood ratio. | | | | | | | | |  |

| **Supplementary Table 6.** A) Diagnostic performance of CoDE-HF rule-out score | | | | | | | | |  |  |
| --- | --- | --- | --- | --- | --- | --- | --- | --- | --- | --- |
| **Age group** | **True positive** | **False positive** | **True negative** | **False negative** | **Negative predictive value**  **(95% CI)** | **Sensitivity**  **(95% CI)** | **LR-**  **(95% CI)** | **Proportion ruled out**  **(%)** | | |
| <50 | 211 | 172 | 954 | 5 | 99.5  (98.8-99.8) | 97.7  (94.6-99.0) | 0.011  (0.004-0.032) | 71.5 | | |
| 50 - 59 | 355 | 332 | 590 | 7 | 98.8  (97.6-99.4) | 98.1  (96.0-99.1) | .025  (0.015-0.034) | 46.5 | | |
| 60 - 69 | 688 | 544 | 520 | 10 | 98.2  (96.0-99.2) | 98.7  (96.6-99.5) | 0.027  (0.009-0.078) | 30.1 | | |
| 70 - 79 | 1204 | 880 | 322 | 12 | 96.4  (93.8-97.9) | 99.1  (98.0-99.6) | 0.024  (0.008-0.069) | 13.8 | | |
| ≥80 | 1594 | 819 | 106 | 2 | 98.1  (92.9-99.5) | 99.9  (99.0-100.0) | 0.006  (0.002-0.016) | 4.3 | | |
| Overall | 4052 | 2747 | 2492 | 36 | 98.5  (97.7-99.1) | 99.2  (98.5-99.6) | 0.023  (0.012-0.043) | 27.1 | | |
| CI: confidence interval; LR-: negative likelihood ratio. | | | | | | | | | |  |

| B) Diagnostic performance of CoDE-HF rule-in score | | | | | | | | |  |
| --- | --- | --- | --- | --- | --- | --- | --- | --- | --- |
| **Age group** | **True positive** | **False positive** | **True negative** | **False negative** | **Positive predictive value**  **(95% CI)** | **Specificity**  **(95% CI)** | **LR+**  **(95% CI)** | **Proportion ruled in**  **(%)** | |
| <50 | 123 | 27 | 1099 | 93 | 82.0  (75.0-87.4) | 97.9  (95.7-99.0) | 20.4  (10.1-40.9) | 11.2 | |
| 50 - 59 | 202 | 50 | 872 | 160 | 81.1  (74.7-86.2) | 95.0  (92.0-96.9) | 10.5  (5.9-18.6) | 19.6 | |
| 60 - 69 | 395 | 87 | 977 | 303 | 82.1  (75.3-87.3) | 93.2  (89.0-95.9) | 6.2  (3.8-10.2) | 27.4 | |
| 70 - 79 | 786 | 142 | 1060 | 430 | 84.2  (75.1-90.4) | 90.2  (84.2-94.0) | 5.0  (3.1-7.9) | 38.4 | |
| ≥80 | 1145 | 200 | 725 | 451 | 83.2  (76.1-88.5) | 80.8  (73.8-86.4) | 2.6  (1.9-3.5) | 53.4 | |
| Overall | 2651 | 506 | 4733 | 1437 | 83.4  (75.2-89.2) | 91.5  (87.6-94.3) | 5.9  (4.1-8.3) | 33.8 | |
| CI: confidence interval; LR+: positive likelihood ratio. | | | | | | | | |  |

**Supplementary Figure 1. Negative likelihood ratio of NT-proBNP rule-out threshold of 300 pg/mL and CoDE-HF low probability score.**


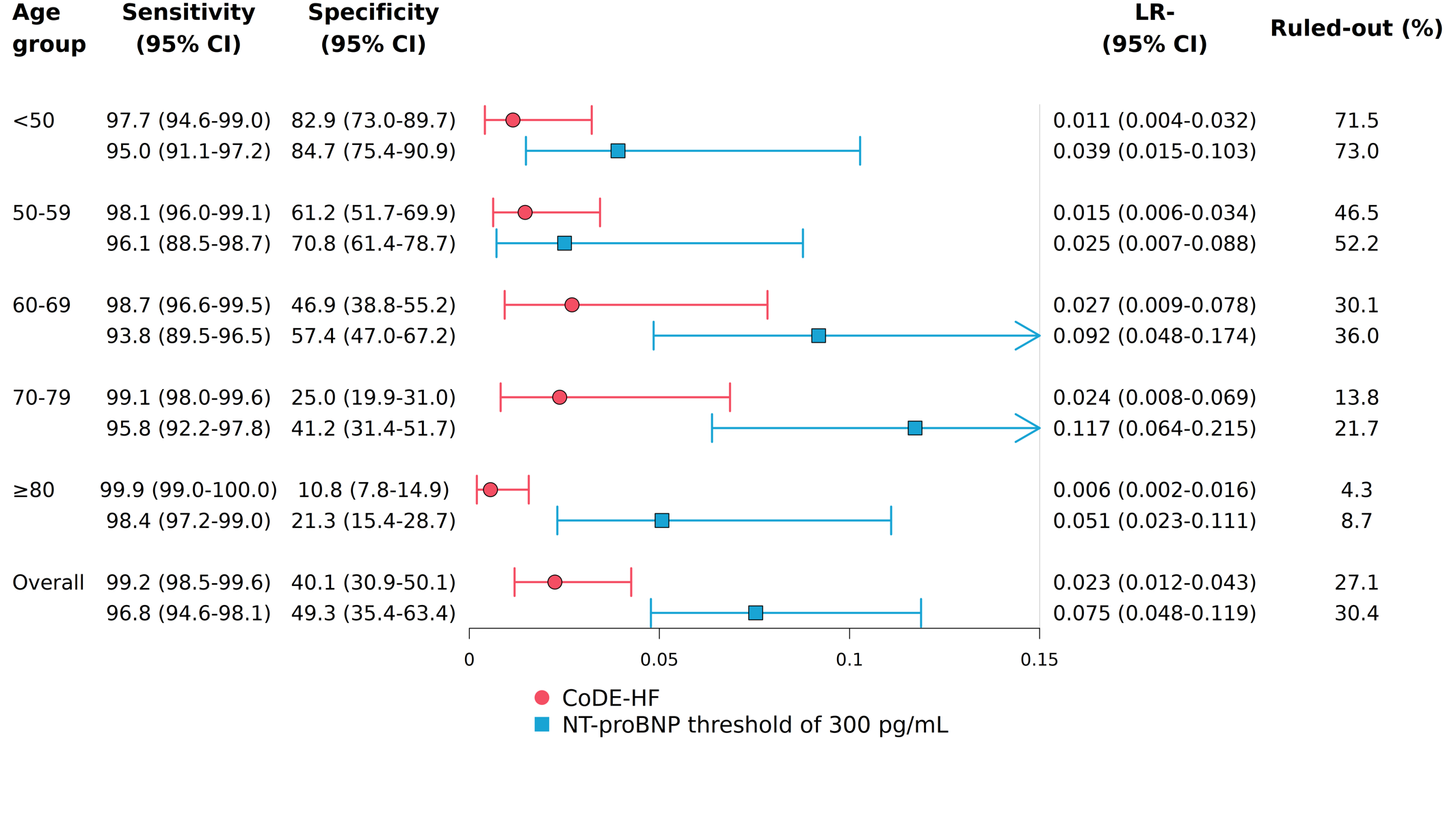


**Supplementary Figure 2. Negative predictive value of NT-proBNP thresholds of 100, 200, and 300 pg/mL and CoDE-HF low-probability score.**


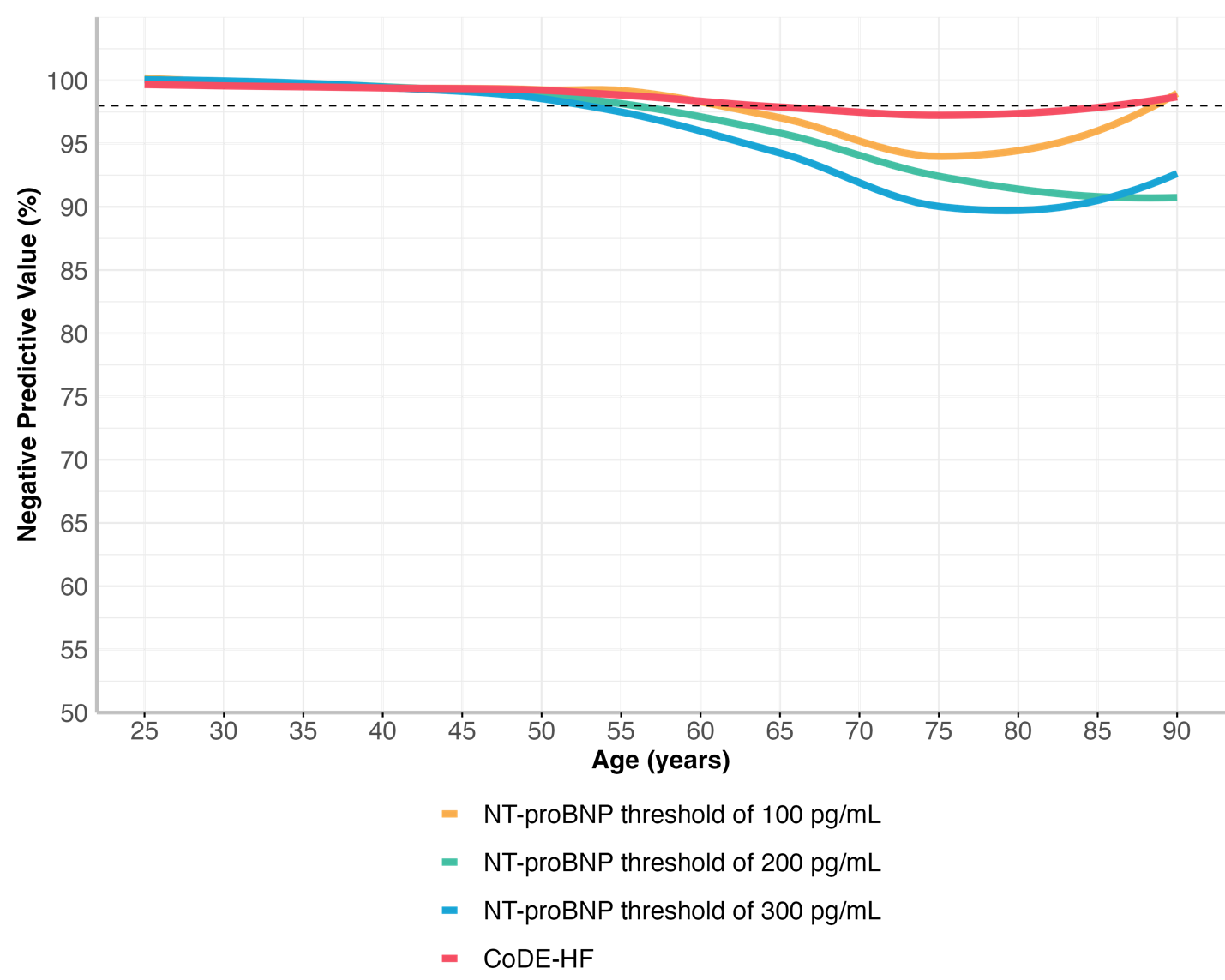


**Supplementary Figure 3. Positive likelihood ratio of NT-proBNP age-specific rule-in thresholds and CoDE-HF high probability score.** (Age-specific NT-proBNP thresholds: 450, 900, and 1,800 pg/mL for those <50 years, 50-75 years, and >75 years, respectively)


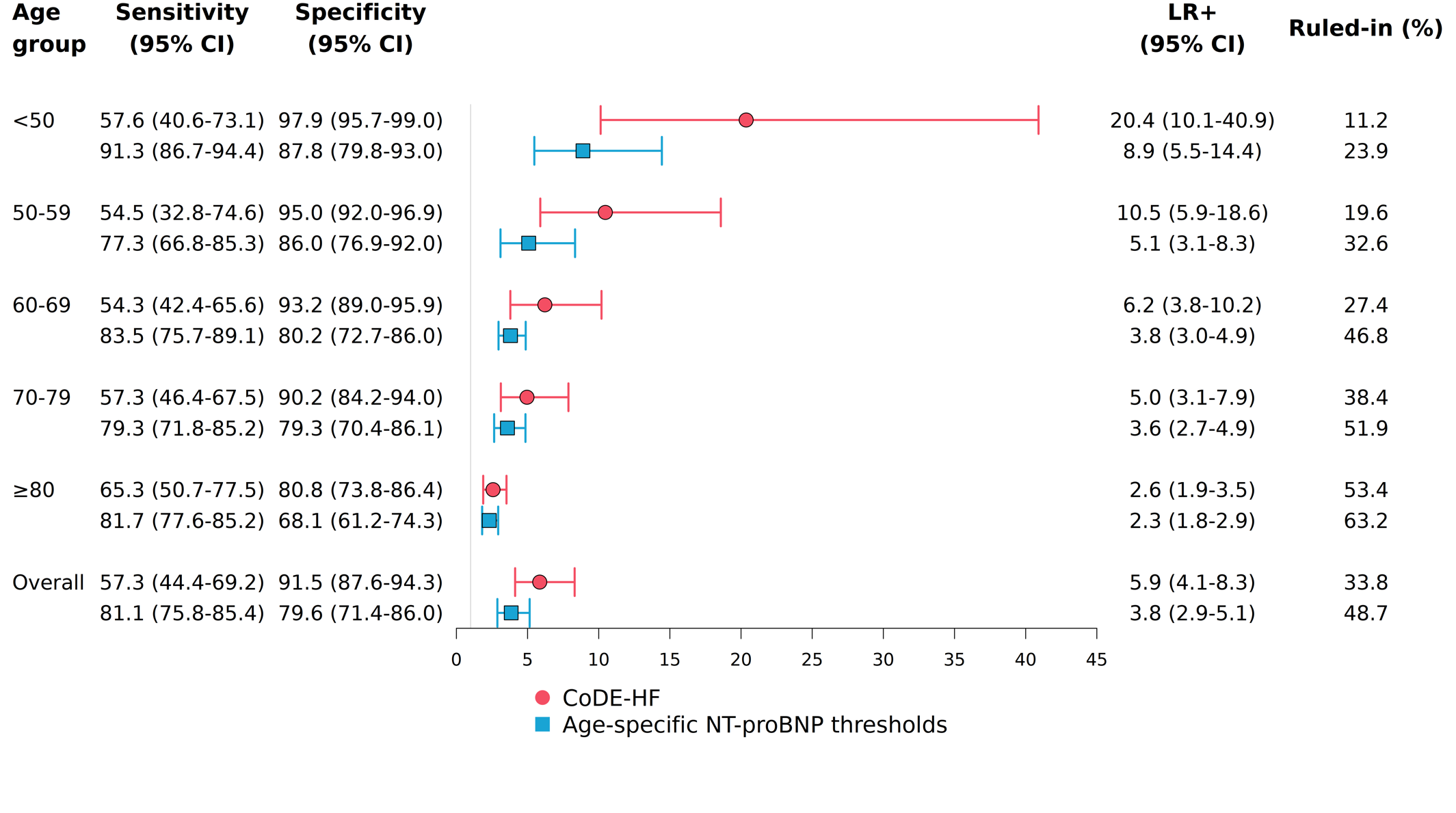


**Supplementary Figure 4. Negative predictive value of NT-proBNP rule-out threshold of 300 pg/mL and CoDE-HF low-probability score in patients without prior heart failure.**

1. Negative predictive value across age as a continuous variable

**
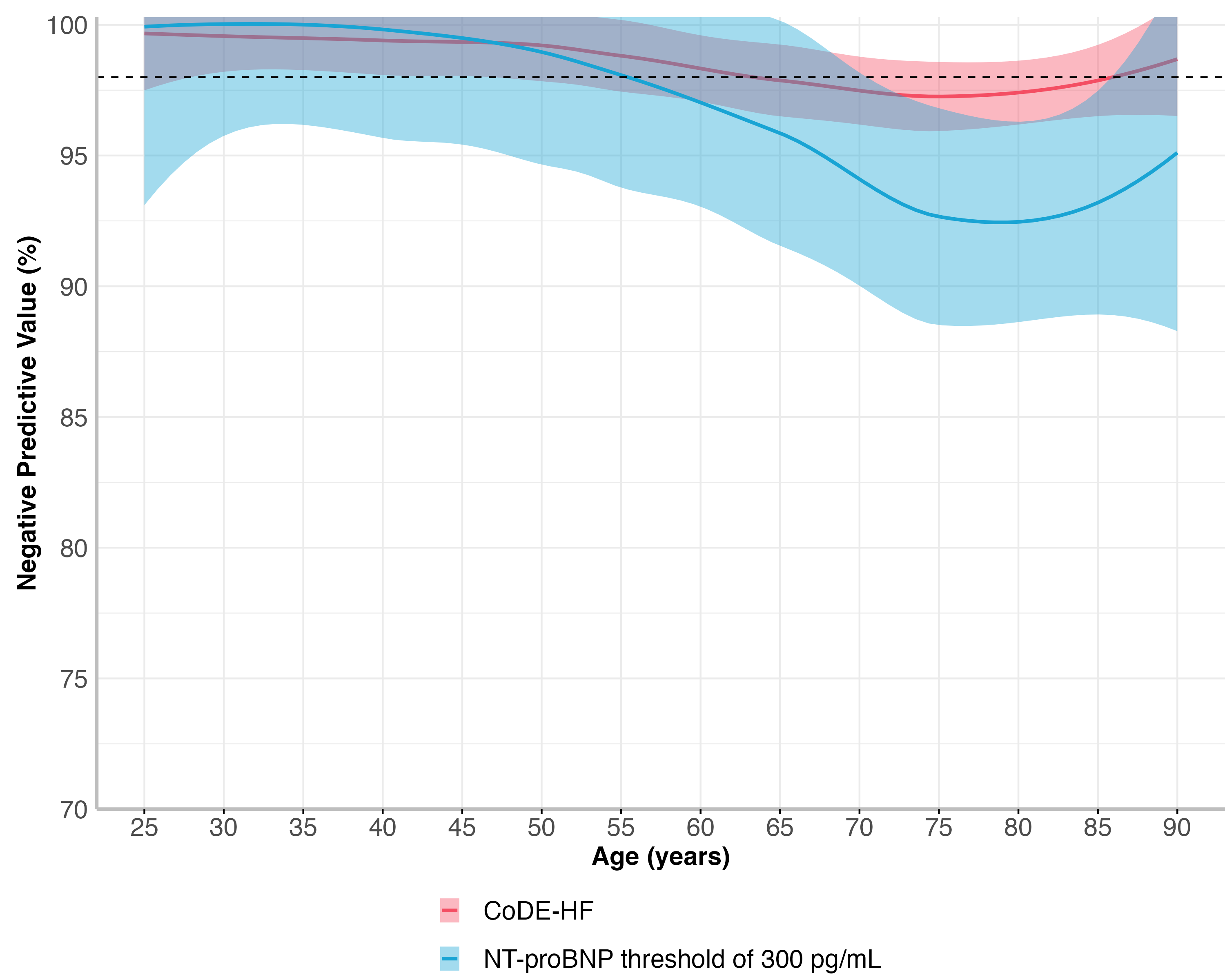
**

1. Forest plot of negative predictive value across patient age groups


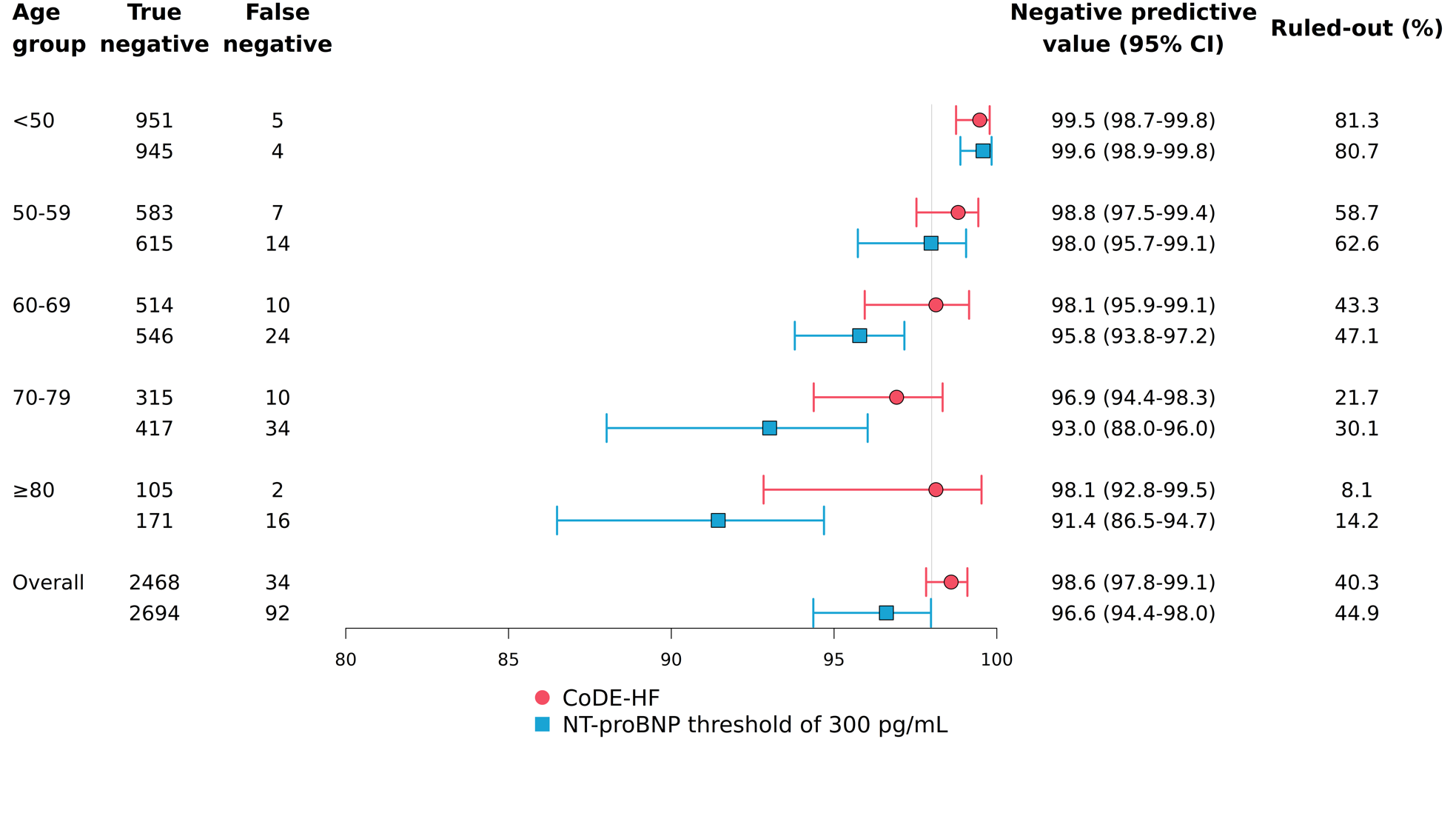


**Supplementary Figure 5. Positive predictive value of age-specific NT-proBNP rule-in thresholds and CoDE-HF high-probability score in patients without prior heart failure.**

(Age-specific NT-proBNP thresholds: 450, 900, and 1,800 pg/mL for those <50 years, 50-75 years, and >75 years, respectively)

A) Positive predictive value across age as a continuous variable


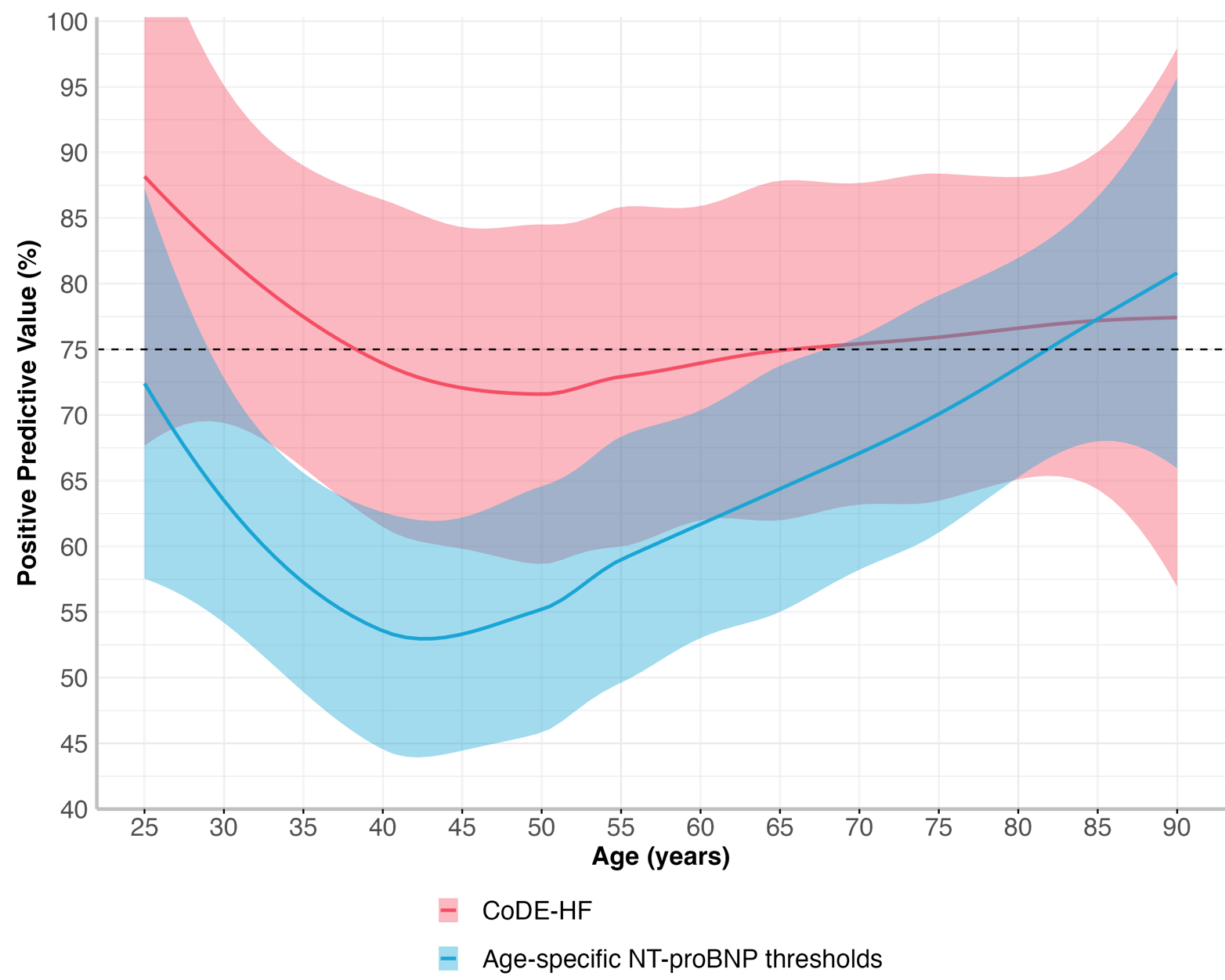


1. Forest plot of positive predictive value across patient age groups

**
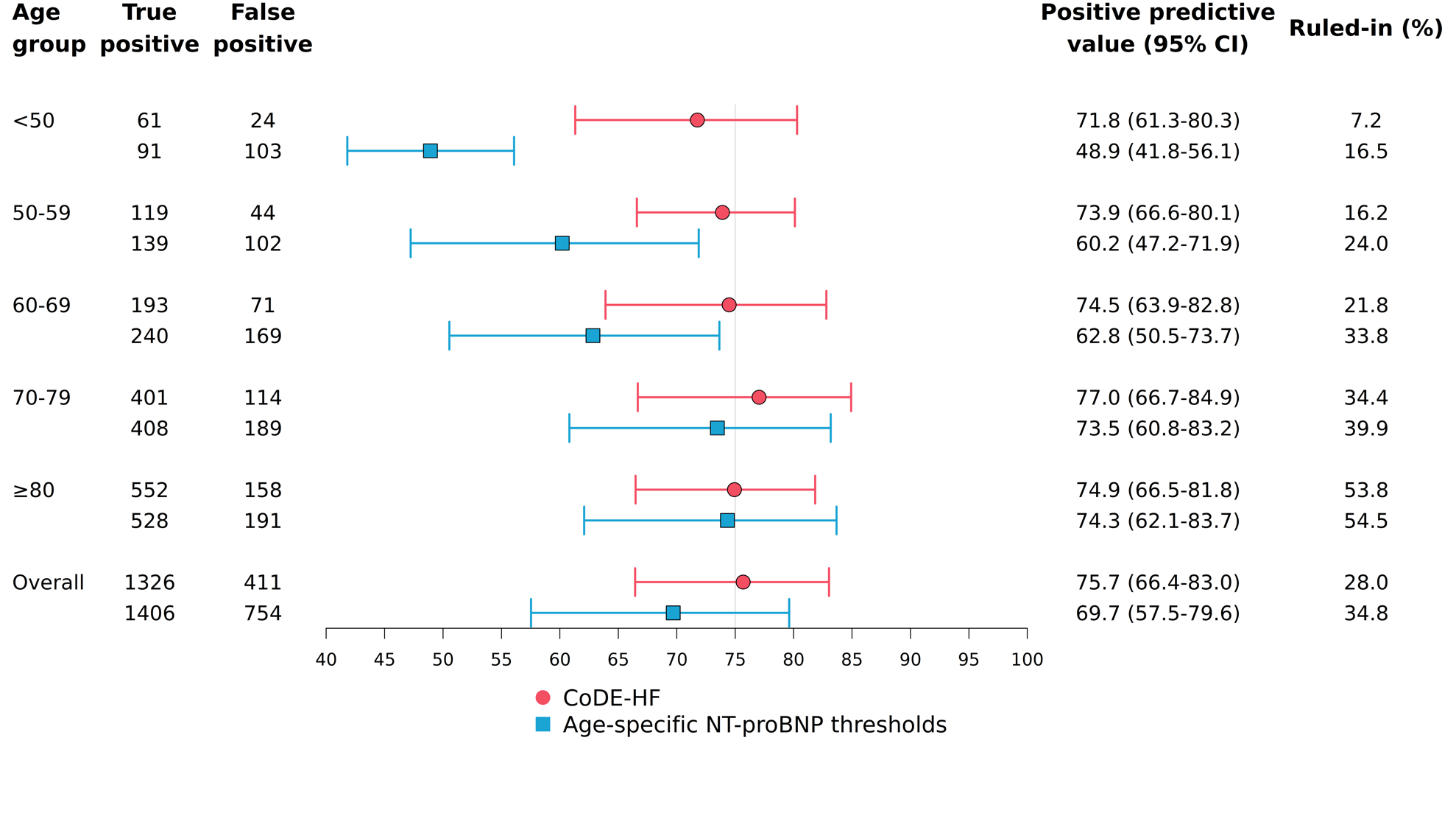
**

**Supplementary Figure 6.** **Discrimination of NT-proBNP and CoDE-HF for the diagnosis of acute heart failure in patients without prior heart failure.**

A) Receiver operator curve of NT-proBNP stratified by age groups


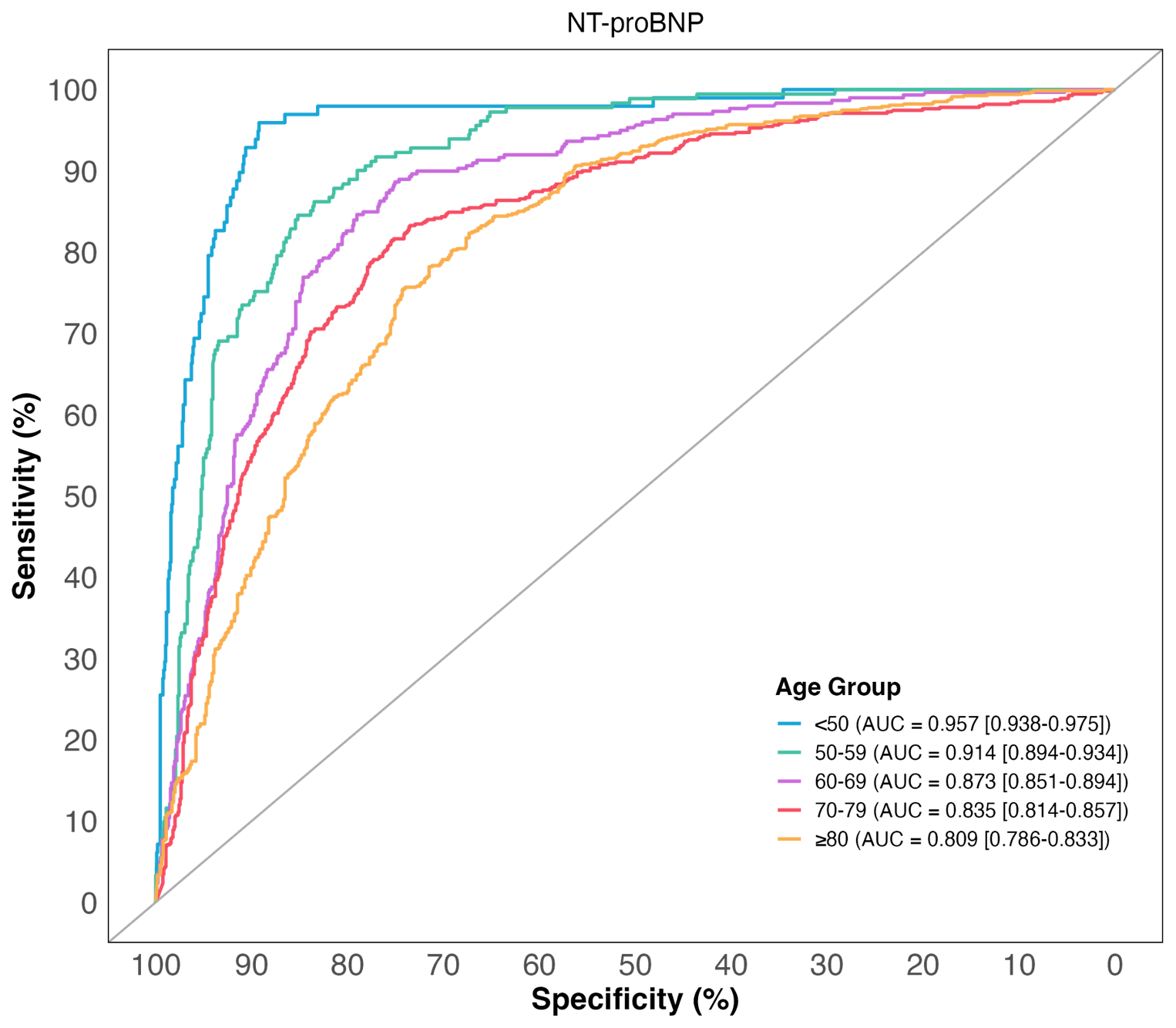


B) Receiver operator curve of CoDE-HF stratified by age groups


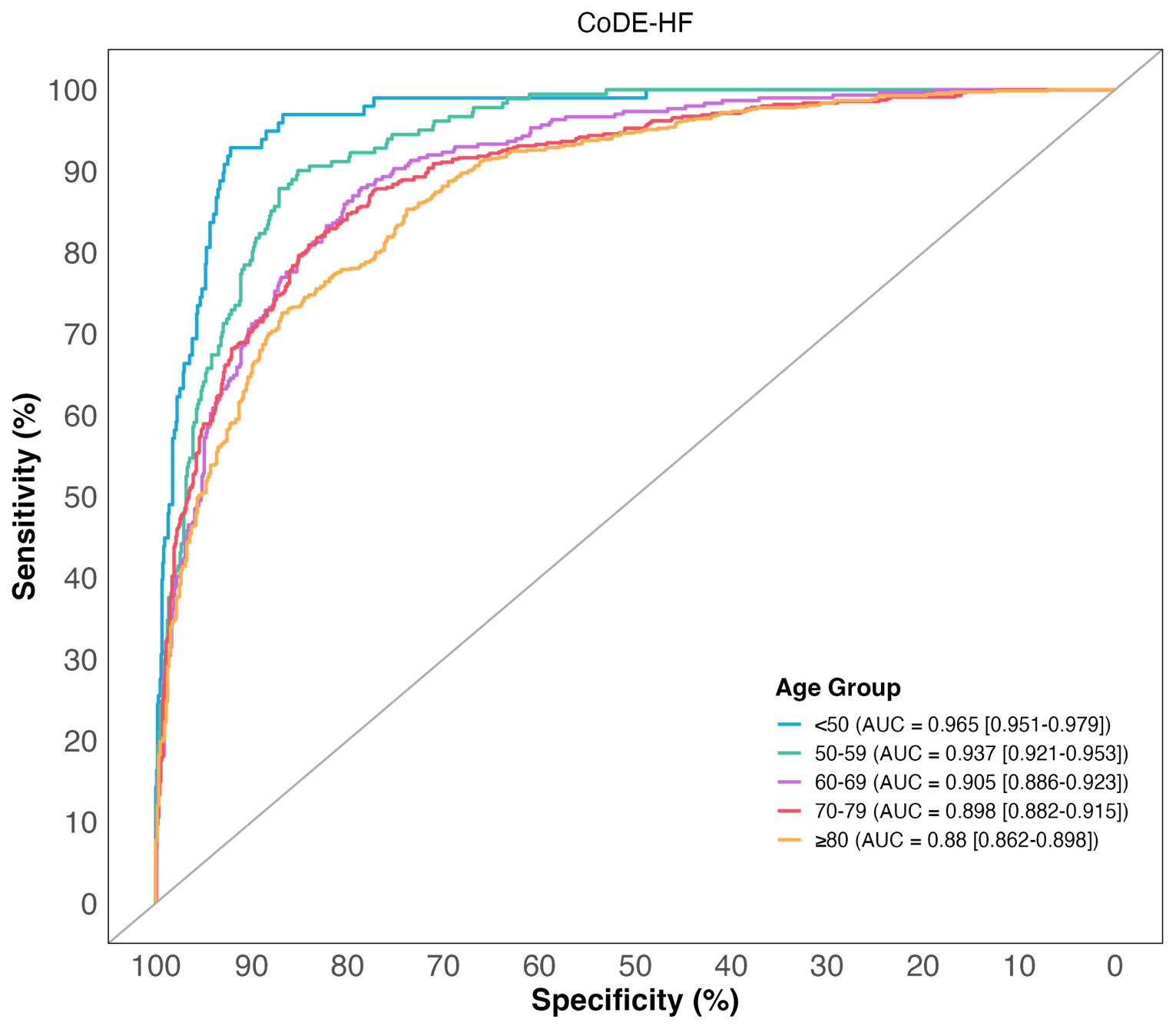

Supplement: xvaf006_Supplementary_Data [file xvaf006_supplementary_data.docx]
